# Exploring the mechanisms of baicalein in lung adenocarcinoma: an integrative preliminary study with focused experimental assessment in A549 cells

**DOI:** 10.3389/fonc.2026.1912108

**Published:** 2026-10-06

**Authors:** Jiaqian Yuan, Jiayan Zhong, Ruilin Chen, Jia Mao, Zhen Wang

**Affiliations:** 1 The First School of Clinical Medicine, Zhejiang Chinese Medical University, Hangzhou, China; 2 Department of Respiratory and Critical Care Medicine, The First Affiliated Hospital of Zhejiang Chinese Medical University, Hangzhou, China; 3 Department of Respiratory and Critical Care Medicine, Ningbo TCM Hospital Affiliated to Zhejiang Chinese Medical University, Ningbo, China

**Keywords:** AKT1, baicalein, BCL2, immune microenvironment, integrative study, lung adenocarcinoma

## Abstract

**Background:**

Lung adenocarcinoma (LUAD) continues to present therapeutic challenges because of pathway redundancy and treatment resistance, underscoring the need for multi-target and systems-level strategies. Baicalein (BA), a flavonoid metabolite from *Scutellaria baicalensis* Georgi, shows antitumor potential, yet integrative and immune-related evaluations of BA in LUAD remain limited.

**Methods:**

We integrated network analyses with TCGA-LUAD transcriptomics, weighted gene co-expression network analysis (WGCNA), network topology, GO/KEGG enrichment, bulk RNA-seq-based immune-infiltration estimation (CIBERSORT), molecular docking, and clinical association analysis (GEPIA2/HPA). Cell viability was assessed in A549 cells​ using the CCK-8 assay, and AKT1 phosphorylation and BCL2 protein expression were further examined experimentally by western blot analysis.

**Results:**

A total of 117 candidate BA-LUAD overlapping targets were identified, and six candidate hub genes (EGFR, AKT1, TP53, BCL2, HIF1A, and ESR1) were prioritized. Enrichment analyses suggested that the candidate network was associated with oncogenic signaling and stress-response programs, particularly PI3K-AKT/MAPK signaling, ROS-related processes, apoptosis, and p53 signaling. CCK-8 assays showed that BA reduced A549 cell viability *in vitro*, with IC_50_ values of 54.93 μM at 24 h and 22.83 μM at 48 h. Among the prioritized candidates, AKT1 and BCL2 were selected for focused experimental assessment in A549 cells. BA treatment reduced AKT1 phosphorylation and BCL2 protein expression in A549 cells, whereas total AKT1 remained largely unchanged. HIF1A expression showed broad associations with computationally estimated immune-cell fractions and poorer overall survival.

**Conclusion:**

These findings prioritize a BA-associated candidate network in LUAD involving kinase signaling, apoptosis-related regulation, oxidative-stress responses, and immune-microenvironment associations. The CCK-8 results support an inhibitory effect of BA on A549 cell viability *in vitro*, and the observed changes in AKT1 phosphorylation and BCL2 protein expression provide focused experimental support for selected components of the candidate network; further *in vitro* and *in vivo* studies are required to validate biological relevance and translational potential.

## 1 Introduction

Lung cancer is an aggressive malignancy, ranking among the most commonly diagnosed cancers and serving as the leading cause of cancer-related mortality worldwide (1). Among its subtypes, lung adenocarcinoma (LUAD) is currently the most prevalent form of primary lung cancer, accounting for approximately 40%-45% of all cases (2). Despite significant advances in the treatment of LUAD, challenges such as treatment-related adverse effects, drug resistance, and poor long-term survival persist (3). In particular, although the development of epidermal growth factor receptor tyrosine kinase inhibitors (EGFR-TKIs) has improved prognosis in a subset of LUAD patients, therapeutic resistance frequently emerges through adaptive signaling pathways, remodeling of the tumor microenvironment (TME), and other pharmacological resistance mechanisms (4). In fact, most LUAD cases are diagnosed at advanced stages, resulting in a 5-year survival rate of only around 15% (5). The pathogenesis and progression of LUAD involve highly complex and interconnected molecular processes, characterized by extensive pathway diversity, signaling redundancy, and TME complexity (6). Consequently, sustained therapeutic efficacy is difficult to achieve through modulation of a single pathway alone. This complexity underscores the necessity of system-level, multi-target therapeutic strategies capable of simultaneously regulating oncogenic signaling networks and the immune microenvironment. Accordingly, there is an increasing demand for novel multi-target therapeutic approaches with favorable safety profiles, particularly those derived from natural products.

Traditional Chinese Medicine (TCM), a medical system with millennia of clinical practice, includes numerous botanical medicines containing bioactive flavonoid metabolites with pharmacological potential. Many TCM-derived agents have been shown to enhance the efficacy of conventional therapies, reduce adverse effects, modulate the immune system, reverse drug resistance, and inhibit metastasis through multiple mechanisms (7). Baicalein (BA), a flavonoid metabolite extracted from *Scutellaria baicalensis* Georgi, exhibits various pharmacological activities, including anti-inflammatory, antioxidant, and antitumor effects. Previous studies have indicated that BA possesses significant antitumor potential in multiple tumor models, mainly by inducing apoptosis, inducing cell-cycle arrest, modulating autophagy and metabolism, and overcoming drug resistance. (3, 8). Moreover, BA has also been reported to modulate immune cells, endothelial cells, fibroblasts, and extracellular matrix components within the TME, thereby inhibiting tumor angiogenesis, progression, and metastasis (9). Indeed, accumulating experimental evidence has also demonstrated that BA exerts inhibitory effects in lung cancer models, including suppression of LUAD cell proliferation, migration, and vasculogenic mimicry through modulation of oncogenic signaling pathways and tumor-associated microenvironmental processes (10–12).

However, despite its reported anticancer activity, the disease-related candidate mechanisms and molecular targets of BA in LUAD remain insufficiently understood. Most existing studies have focused on isolated targets or individual signaling pathways, which limits a comprehensive understanding of its context-specific biological actions. Notably, few integrative systems-level and immune-related evaluations of BA in LUAD have been reported. Network analysis and bioinformatics provide a hypothesis-generating framework for prioritizing candidate targets and pathways of natural flavonoid metabolites, and molecular docking can provide preliminary structural indications of ligand-protein compatibility (13, 14). To extend existing network pharmacology approaches, this study integrated predicted BA-associated targets with LUAD transcriptomic alterations, co-expression modules, immune-microenvironmental features, clinical associations, molecular docking, and focused cell-based assessment. This cross-layer strategy was designed to improve the disease-specific prioritization of candidate mechanisms and generate more focused hypotheses for subsequent investigation.

## 2 Materials and methods

### 2.1 Identification of potential targets of BA

First, the chemical structure of BA was retrieved from the PubChem database (https://pubchem.ncbi.nlm.nih.gov/). Potential protein targets of BA were predicted using TCMSP (OB≥30%, DL≥0.18; https://old.tcmsp-e.com/tcmsp.php), PharmMapper (Norm Fit≥0.4, Human protein targets only; http://www.lilab-ecust.cn/pharmmapper/), and Swiss Target Prediction (Probability≥0.01, Homo sapiens; http://www.swisstargetprediction.ch/). Gene symbols for the predicted targets were standardized via the UniProt database (http://www.uniprot.org/). The targets from all three databases were merged, and duplicates were removed to determine the final set of BA targets.

### 2.2 Identification of LUAD-related targets

Known LUAD-related targets were retrieved from OMIM (https://omim.org/), GeneCards (https://www.genecards.org/), and CTD (https://ctdbase.org/) databases using the keywords “lung adenocarcinoma” and “Adenocarcinoma of Lung.” To ensure the validity of the disease targets selected for further study, targets were filtered based on scores (GeneCards: relevance scores≥10; CTD: inference score≥20). These thresholds were selected to prioritize targets with stronger disease associations and to reduce weakly supported or nonspecific entries, consistent with previous studies that applied score-based filtering strategies using GeneCards and CTD databases (15, 16).

To comprehensively identify LUAD-related targets, RNA-seq data from the TCGA-LUAD cohort were analyzed through differential gene expression analysis and weighted gene co-expression network analysis (WGCNA). Differentially expressed genes (DEGs) between tumor and normal tissues were obtained according to the criteria of false discovery rate (FDR, adjusted by the Benjamini-Hochberg (BH) method) < 0.05 and | log2 (fold change) | > 2 using the “*edgeR*” package in R. The results were visualized as a volcano plot using the “*ggplot2*” package.

The “*WGCNA*” package in R was used to construct a co-expression network to identify gene modules associated with LUAD clinical information. A total of 598 TCGA-LUAD samples, including 539 LUAD and 59 normal samples, were included in the analysis. The data preprocessing included log2 transformation, filtering low-variance genes (standard deviation < 0.5), and sample clustering to ensure data quality. Hierarchical sample clustering was performed to identify potential sample outliers. No obvious outlier samples were detected, and all 598 samples were retained for subsequent network construction. An appropriate soft-thresholding power (β = 9) was determined based on the scale-free topology criterion, corresponding to a scale-free topology fit index of R^2^ = 0.90 while retaining adequate mean connectivity, followed by the construction of a weighted adjacency matrix and transformation into a topological overlap matrix (TOM). Hierarchical clustering was performed using the “hclust” algorithm combined with dynamic tree cutting (minModuleSize = 50, deepSplit = 2) to identify co-expression modules. Modules with high similarity (MEDissThres < 0.25) were merged. Pearson correlation analysis was conducted to calculate the correlation coefficients and p-values between the module eigengenes (MEs) and external clinical information. Key genes within critical modules were selected based on Module Membership > 0.8 and Gene Significance > 0.5. A formal module-preservation analysis was not performed in the present study.

To control potential noise and false positives in disease-related gene identification, LUAD-associated targets were screened using stringent and complementary criteria. DEGs were first intersected with WGCNA-identified key genes to identify candidates supported by complementary LUAD transcriptomic and co-expression evidence. These genes were then integrated with LUAD-related targets collected from the three aforementioned databases using strict relevance and inference score thresholds, followed by deduplication to generate a comprehensive disease-associated gene set. Subsequently, the resulting LUAD-related genes were intersected with predicted BA-associated targets to identify candidate BA-LUAD overlapping targets for downstream analyses.

### 2.3 Construction of protein-protein interaction (PPI) network and identification of hub genes

The 117 overlapping target genes identified in the previous step were uploaded to the STRING database 12.0 (https://cn.string-db.org/) to generate a PPI network under the “Homo sapiens” setting. The minimum required interaction score was set to high confidence (0.700), and disconnected nodes were excluded. The predicted network of the top 20 nodes selected according to network topological ranking was visualized using Cytoscape 3.10.3.

In the PPI network, hub genes were identified using the cytoNCA plugin based on various topological features: Betweenness Centrality (BC), Degree Centrality (DC), Closeness Centrality (CC), Eigenvector Centrality (EC), and Local Average Connectivity (LAC). Initially, the median value of each topological feature was calculated as a threshold. Genes exceeding all median thresholds were retained, and the process was repeated iteratively until fewer than 10 hub genes remained. To computationally cross-check the network-topology-based prioritization, the top 10 hub genes identified by the cytoHubba plugin’s Maximal Clique Centrality (MCC) algorithm were cross-referenced with the cytoNCA-derived candidates. The final candidate hub genes were those that met both the cytoNCA iterative filtering criteria and the top 10 genes identified by the MCC algorithm in the cytoHubba plugin.

Additionally, the MCODE plugin was used to further explore potential functional modules. Protein clusters were obtained using Degree cutoff = 2 and K-Core = 3, followed by visualization.

### 2.4 Enrichment analysis

The Gene Ontology (GO) and Kyoto Encyclopedia of Genes and Genomes (KEGG) enrichment analyses were performed on the 117 target genes using the “*clusterProfiler*” package in R. The GO analysis covered three categories: Biological Process (BP), Molecular Function (MF), and Cellular Component (CC). A significance threshold of adjusted *p*-value < 0.05 was applied for filtering (BH method). To further improve pathway-level interpretability, the pathway which showed high relevance in the enrichment analysis was visualized using the SRplot (https://www.bioinformatics.com.cn/) (17), with gene-level changes mapped onto the corresponding KEGG pathway diagram. Subsequent KEGG analysis was conducted on the high-scoring protein clusters obtained in Section 3.3, using the same BH-adjusted *p*-value < 0.05 as the significance cutoff. The results were visualized using the “*ggplot2*” package.

### 2.5 Construction of target-BP/KEGG network

To clarify the relationship between hub genes and enriched pathways, and to further characterize candidate biological contexts within the BA-LUAD overlapping target network, the top 5 GO-BP terms and their associated genes were visualized as chord diagrams using Sangerbox Tools (http://www.sangerbox.com/tool) (18). For better visualization of the relationships between target genes and KEGG pathways related to LUAD, unrelated pathways were excluded, and a Sankey bubble diagram was created using the SRplot. Additionally, to systematically quantify the distribution of target genes across key biological processes and pathways, the occurrence frequency of each gene in the enriched GO-BP terms and KEGG pathways was calculated. Proportion was defined as (occurrence frequency/total number of terms) × 100%. The 16 most frequently involved genes were presented in tabular format for further interpretation.

### 2.6 Immune infiltration analysis

To explore immune microenvironmental patterns potentially associated with BA-related targets in LUAD, the Cell-type Identification by Estimating Relative Subsets of RNA Transcripts (CIBERSORT) algorithm was applied to estimate the relative fractions of 22 immune cell types in TCGA-LUAD samples. The estimated immune-cell fractions in LUAD tissues were compared with those in normal lung tissues to identify differences in immune-cell composition between the two groups using the two-sided Wilcoxon rank-sum test. Spearman correlation analysis was then used to evaluate the correlation between the expression of six hub genes and immune cell infiltration levels, with results visualized as a heatmap. No correction for multiple comparisons was applied to the immune-cell comparisons or hub gene-immune cell correlation analyses; therefore, the reported p-values are nominal p-values. To provide a complementary computational cross-check, TIMER analysis (https://compbio.cn/timer1/) was further performed. These analyses provide an overview of immune cell infiltration patterns associated with BA-related targets in LUAD.

### 2.7 Molecular docking analysis

Molecular docking was performed using AutoDock Tools 1.5.7 and AutoDock Vina 1.2.7 to assess whether BA could adopt computationally plausible poses within the selected structures of the six hub proteins. The 3D crystal structures of the proteins were downloaded from the Protein Data Bank (PDB, http://www.rcsb.org/), saved in PDB format, and processed using PyMOL 3.1 (a powerful molecular visualization software) to remove water molecules and ligands before uploading to AutoDock Tools for hydrogenation. These proteins were then saved in PDBQT format as receptors. The structure of BA, obtained from the PubChem database, was saved in MOL2 format, imported into AutoDockTools for hydrogenation, and selected as the ligand, then exported as a PDBQT file. Molecular docking was performed using AutoDock Vina 1.2.7, with five docking repetitions and the median binding energy calculated. A docking energy threshold of ≤ -7.0 kcal/mol was interpreted as relatively more favorable predicted structural compatibility, and the results were visualized using PyMOL. To contextualize the docking scores of BA, known inhibitors were included as positive controls and docked using the same receptor structures and docking workflow. MK-2206, ABT-199, gefitinib, tamoxifen, and YC-1 were used as reference compounds for AKT1, BCL2, EGFR, ESR1, and HIF1A, respectively. No direct positive-control ligand was included for TP53 because TP53 is not a conventional enzymatic target with a well-defined ligand-binding pocket, and commonly used p53-pathway modulators either target the MDM2-p53 interaction or act through mutation-dependent p53 reactivation (19). For protein structures containing suitable co-crystallized ligands, redocking validation was performed by removing the native ligand and docking it back into the original binding pocket using the same docking parameters. The redocked ligand pose was then compared with the original crystallographic ligand pose, and RMSD values were calculated to evaluate whether the docking workflow could reproduce the native binding mode.

### 2.8 Expression and prognostic analysis of hub genes

To characterize the expression patterns and clinical associations of the candidate hub genes in LUAD, multi-dimensional analyses were conducted using the Human Protein Atlas (HPA, https://www.proteinatlas.org/) and GEPIA2 (http://gepia2.cancer-pku.cn/) platforms. First, immunohistochemical images of hub target proteins in normal lung tissues and LUAD tissues were retrieved from the HPA database to compare protein expression levels. Then, mRNA expression differences of hub targets between tumor and normal samples were analyzed using transcriptomic data from GEPIA2, and their expression trends across different tumor stages (I-IV) were assessed. Finally, survival curves were generated using the survival analysis module in GEPIA2 to evaluate the associations between candidate hub-gene expression and overall survival in LUAD patients.

### 2.9 Chemicals and reagents

Baicalein (purity: 98%, No. 491-67-8) was obtained from Sigma-Aldrich (St. Louis, MO, USA). Cell Counting Kit-8 (CCK-8; No. BS350B) was purchased from Biosharp (Hefei, China). RIPA lysis buffer (No. AR0102), protease inhibitor (No. AR1182) and phosphatase inhibitor (No. AR1183) were purchased from Boster (Wuhan, China). Antibodies including phospho-AKT1 (Ser 473) (No. K10L8), AKT1 (No. C19L11) and GAPDH (No. A19L10), BCL2 (No. H20N17) and β-actin (No. C21N23) were purchased from Selleckchem (Houston, TX, USA). The secondary antibody was an anti-rabbit secondary antibody (No. RS0002) (Immunoway, Plano, TX, USA).

### 2.10 Cell culture

A549 cells were purchased from the Cell Bank of Chinese Academy of Sciences (Shanghai, China) and maintained in F-12K medium supplemented with 10% fetal bovine serum. Cells were cultured in 5% CO_2_ at 37 °C. BA was dissolved in DMSO and stored at -20 °C. The cell line was authenticated by short tandem repeat (STR) profiling, and was routinely confirmed to be mycoplasma-free.

### 2.11 Cell viability assay

Cell viability was assessed using the Cell Counting Kit-8 assay (CCK-8; Biosharp, BS350B). A549 cells were seeded into 96-well plates at a density of 5 × 10^3^ cells per well and allowed to adhere for 24 h. Cells were then treated with BA at 0 (DMSO control), 6.25, 12.5, 25, 50, 100, and 200 μM for 24 or 48 h. After treatment, CCK-8 reagent was added at 10% of the culture volume and incubated for 1 h. The absorbance was then measured at 450 nm using a microplate reader. Cell viability was calculated relative to the DMSO control after background subtraction. Each condition was measured in three technical replicate wells, which were averaged within each independent experiment. Three independent biological experiments were performed on different days using independently seeded and treated cells. IC_50_ values were estimated by nonlinear regression using GraphPad Prism 9.5. For each treatment duration, the biological-replicate response values at each non-zero BA concentration were fitted using a log(inhibitor) versus response three-parameter nonlinear regression model.

### 2.12 Western blot

Based on the CCK-8 results, BA concentrations of 12.5, 25, and 50 μM were selected. Cells treated with an equal volume of DMSO served as the 0 μM control. After 24 h of treatment, A549 cells were lysed with 1 × RIPA lysis buffer containing 1% protease inhibitor and 1% phosphatase inhibitor, and the total protein concentrations were determined. Equal amounts of protein were separated by electrophoresis on 10% SDS-PAGE gels and subsequently transferred onto a PVDF membrane. After blocking, the membranes were incubated with the indicated primary antibodies at 4 °C overnight, followed by incubation with the corresponding secondary antibodies at room temperature for one hour. Proteins were finally detected using enhanced chemiluminescence reagent. Densitometric quantification of protein bands was conducted using ImageJ software, with target protein levels normalized to the loading control. Western blot analyses were performed in three independent biological experiments (n = 3).

### 2.13 Statistical analysis

Statistical analyses were performed using GraphPad Prism 9.5. Experimental data are presented as the mean ± standard deviation (SD) from three independent biological experiments (n = 3). For the CCK-8 assay, concentration-response data were fitted using a log(inhibitor) versus response three-parameter nonlinear regression model, and IC_50_ values are reported. Western blot densitometric data from the control and three BA-treatment groups were analyzed using ordinary one-way analysis of variance (ANOVA), followed by Dunnett’s multiple-comparisons test to compare each BA concentration with the DMSO-treated control. Before ANOVA, normality of the model residuals was assessed using the Shapiro-Wilk test, and homogeneity of variance was evaluated using the Brown-Forsythe test. Neither test indicated a significant violation of the corresponding assumption; therefore, ordinary one-way ANOVA was retained. Given the small number of independent biological experiments (n = 3), however, the resulting parametric inference should be interpreted cautiously. Significance levels are indicated as follows: *p < 0.05, **p < 0.01, and ***p < 0.001.

## 3 Results

### 3.1 Identification of potential targets of BA

Target genes related to BA were predicted using three databases. A total of 37 targets were identified from TCMSP, 157 from PharmMapper, and 38 from SwissTargetPrediction. After merging and removing duplicates, 212 unique predicted BA-associated target genes were obtained. The chemical structure of BA is shown in **Figure 1**, and the predicted BA-associated target genes are listed in **Supplementary Table 1**.

**Figure 1:**
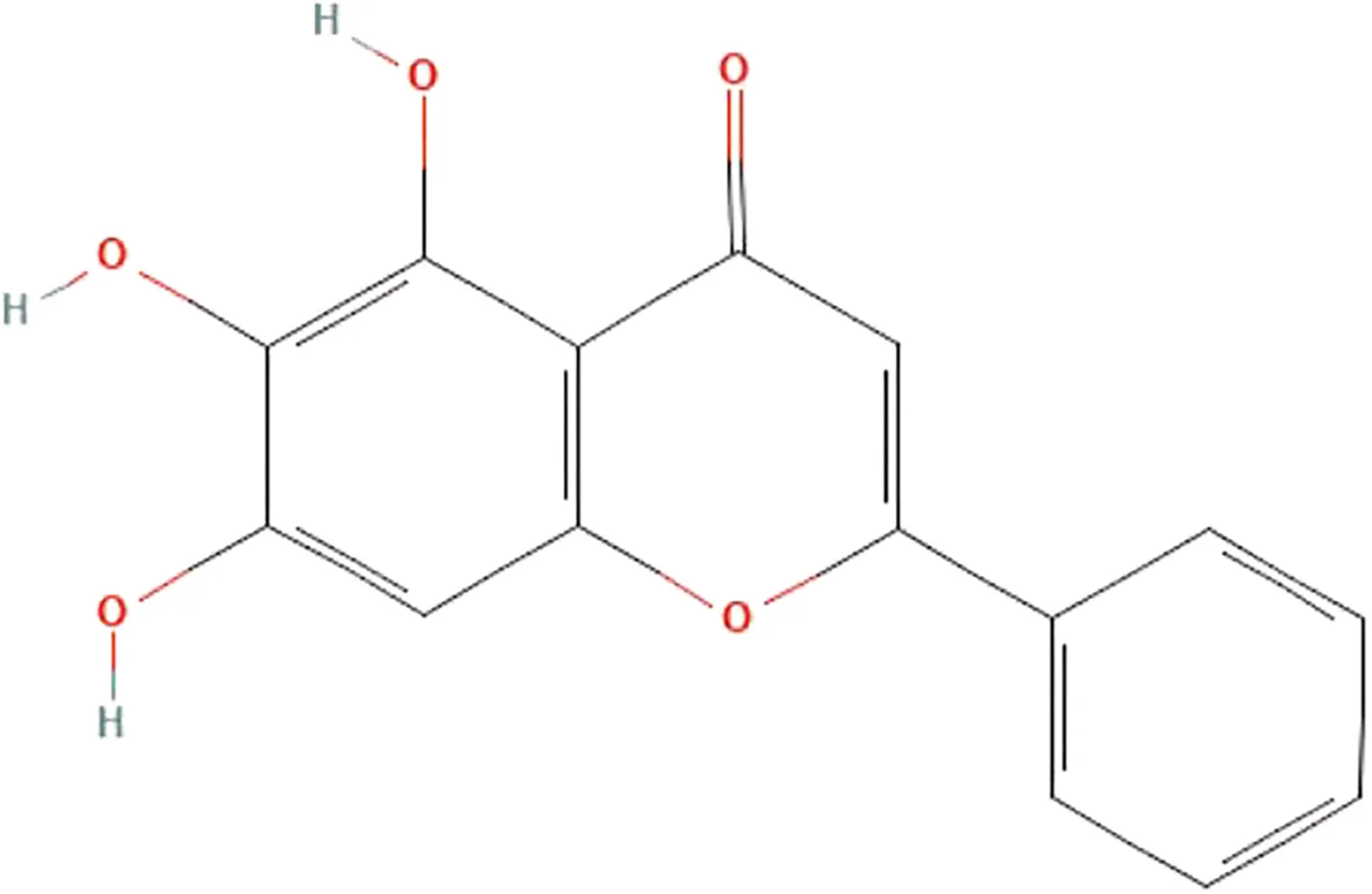
The chemical structure of BA.

### 3.2 Identification of LUAD-related targets

A total of 247 LUAD-related targets were retrieved from OMIM, 2191 from GeneCards, and 42 from the CTD database. After deduplication, 2383 disease targets were compiled (**Supplementary Table 2**).

Data analysis was conducted on selected samples from the TCGA database, including 539 cases of LUAD and 59 normal cases. A total of 2099 DEGs were identified in tumor samples, with 1610 genes upregulated and 489 genes downregulated. The volcano plot is displayed in **Figure 2A**.

**Figure 2:**
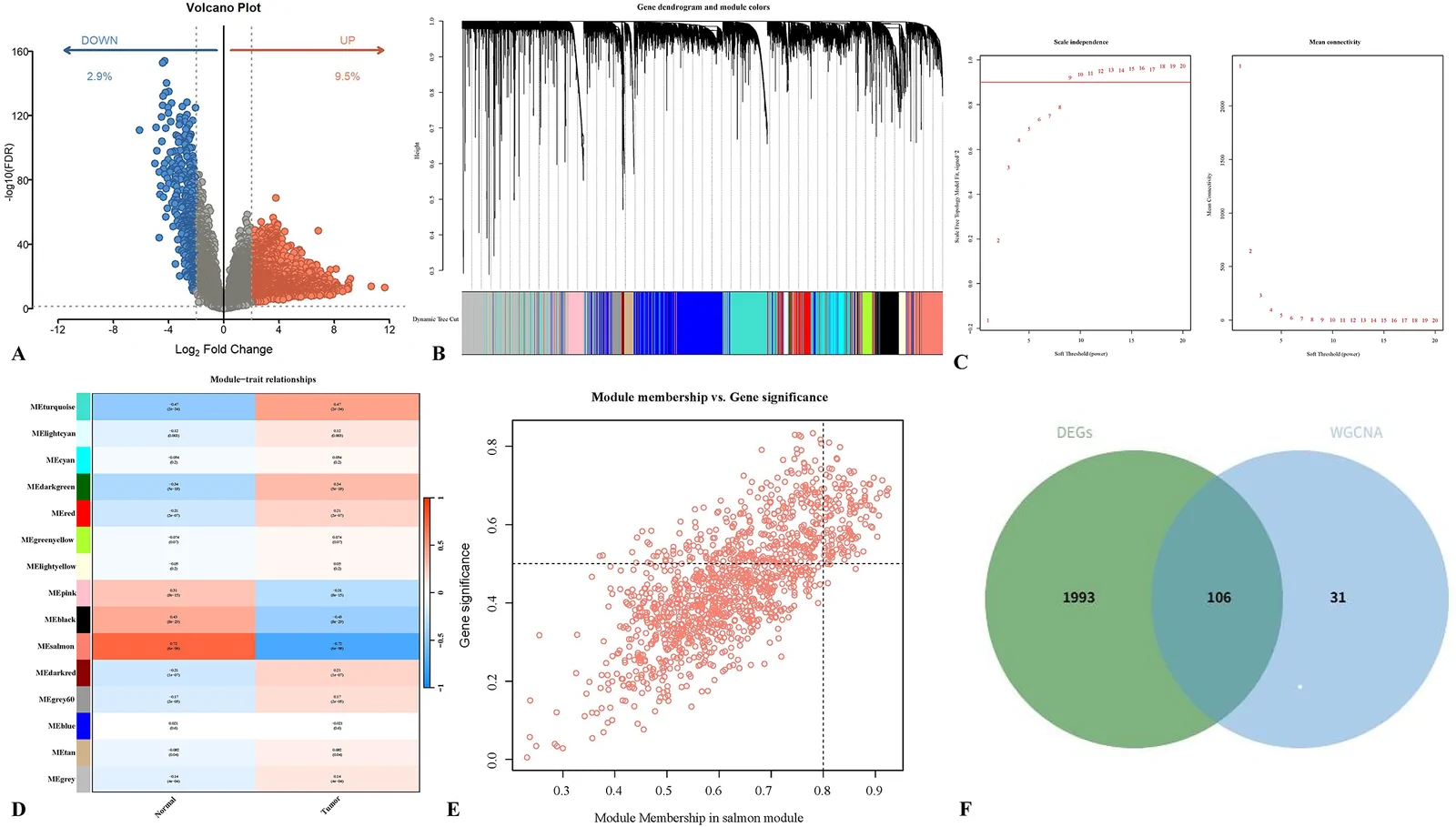
Identification of LUAD-related targets. **(A)** Volcano plot. **(B)** Genes dendrogram. **(C)** Analysis of the optimal soft-thresholding power. **(D)** Heatmap of the relationship between modules and clinical traits. **(E)** Scatter plot of the salmon module. **(F)** Venn diagram of intersection of WGCNA selected key genes and DEGs.

A co-expression network was established via WGCNA methodology, clustering synergistically expressed genes into a gene module (**Figure 2B**). The optimal soft-thresholding power (β = 9) was determined based on scale-free topology fitting (R^2^ = 0.90) and mean connectivity analysis (**Figure 2C**). After merging similar modules, a total of 15 co-expression modules were identified. A heatmap illustrating the relationship between the modules and clinical traits is shown in **Figure 2D**. Among these, the salmon module exhibited the strongest positive correlation with tumor samples (r = 0.72, *p* = 6 × 10^-98^). A scatter plot demonstrated a significant linear correlation between module membership (MM) and gene significance (GS) within this module (**Figure 2E**).

Using strict thresholds, 137 key genes significantly enriched in the salmon module were selected. By intersecting these genes with the 2099 DEGs, 106 candidate LUAD-associated genes supported by both differential-expression and co-expression analyses were identified (**Figure 2F**). Further integration with the 2383 database-derived targets resulted in 2469 consolidated disease targets (**Supplementary Table 3**).

### 3.3 Construction of PPI network and identification of hub genes

The intersection between 212 predicted BA-associated targets and 2469 LUAD-related disease targets yielded 117 candidate BA-LUAD overlapping targets (**Figure 3A**). The top-ranked nodes (n = 23 owing to tied topological rankings) in the PPI network are shown in **Figure 3B**. Node size and color intensity indicate relative topological importance within the predicted PPI network.

**Figure 3:**
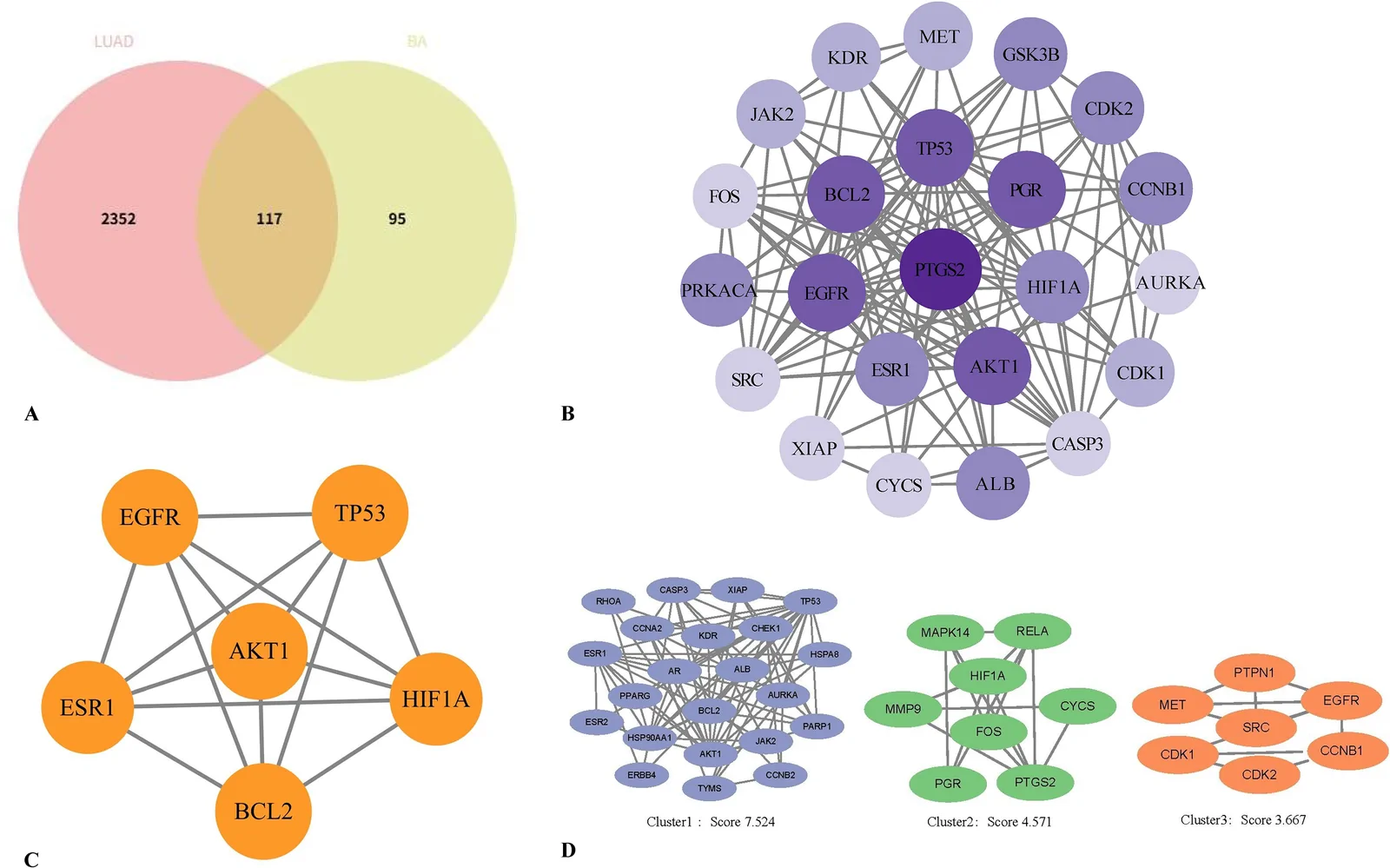
Construction of PPI network and identification of hub genes. **(A)** Venn diagram of intersection of BA and LUAD related targets. **(B)** Simplified PPI network of the top-ranked nodes (23 nodes are shown because several genes shared tied topological rankings at the cutoff). **(C)** Network of hub targets. **(D)** Three protein clusters.

Hub genes were identified using the cytoNCA plugin by selecting nodes with topological parameters (BC, DC, CC, EC, and LAC) greater than the respective median values. After three rounds of filtering, the retained genes were prioritized as candidate hub genes based on their topological properties within the predicted PPI network. This process identified six candidate hub genes within the BA-LUAD overlapping target network, including EGFR, BCL2, AKT1, TP53, ESR1, and HIF1A (**Figure 3C**). These hub genes were further cross-checked using the cytoHubba MCC algorithm, providing complementary computational support for their topological prioritization within the predicted PPI network. The top 10 MCC-ranked genes are listed in **Table 1**.

**Table 1:** Top 10 MCC-ranked genes.

Rank	Name	Score
1	TP53	138075
2	EGFR	128190
3	AKT1	124466
4	BCL2	103596
5	HIF1A	92767
6	SRC	74844
7	FOS	56558
8	PTGS2	56163
9	ESR1	56158
10	MMP9	37154

Three distinct protein clusters were identified by the MCODE plugin (**Figure 3D**), with scores of 7.524, 4.571, and 3.667, respectively. Among the three clusters, Cluster 1 contains the highest concentration of hub targets.

### 3.4 Enrichment analysis

To characterize biological processes and pathways statistically represented within the candidate BA-LUAD target network, GO and KEGG enrichment analyses were performed. GO enrichment analysis identified a total of 1988 statistically significant GO terms, including 1762 terms related to BP, 54 to CC, and 172 to MF (**Supplementary Table 4**). The top 5 significantly enriched terms in each category are visualized in a bubble plot (**Figure 4A**).

**Figure 4:**
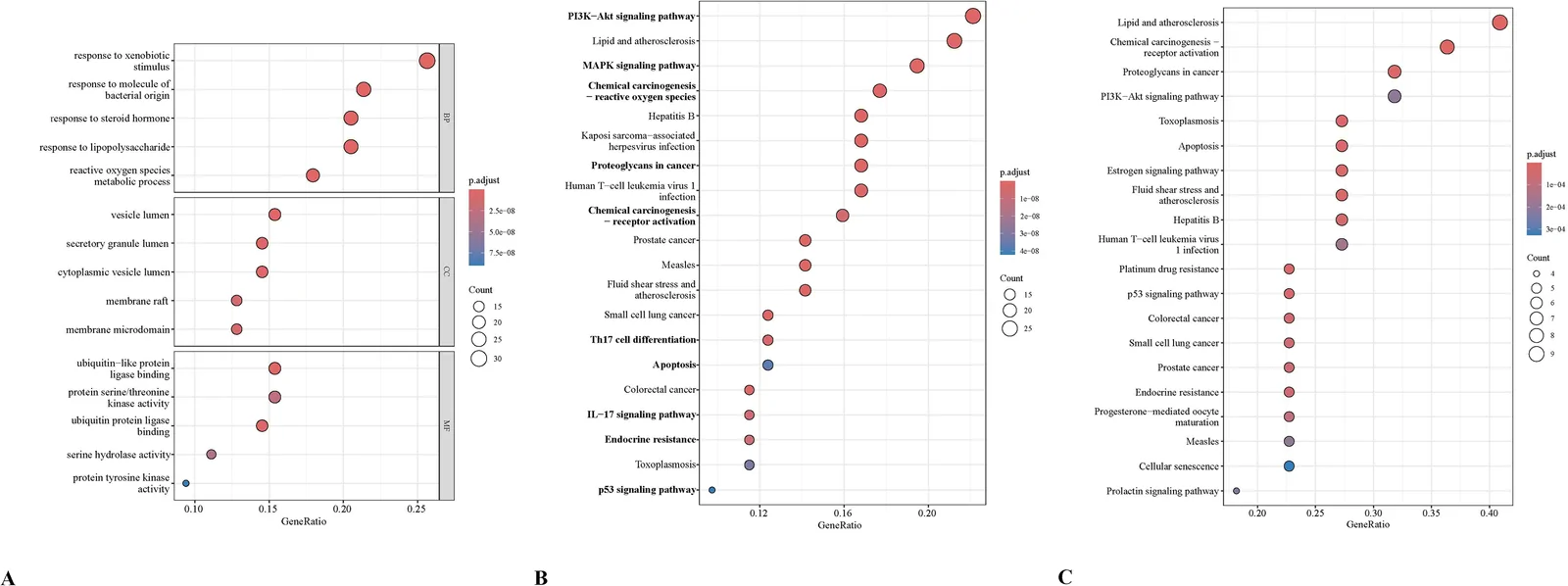
Enrichment analysis. **(A)** Bubble plot of GO terms (top 5). **(B)** Bubble plot of KEGG terms (top 20). **(C)** Bubble plot of KEGG terms for Cluster 1 (top 20).

Among the most relevant BP terms associated with LUAD were response to xenobiotic stimulus, response to molecule of bacterial origin, response to steroid hormone, response to lipopolysaccharide, and reactive oxygen species metabolic process. Enriched CC terms included vesicle lumen, secretory granule lumen, cytoplasmic vesicle lumen, membrane raft, membrane microdomain. The major MF terms were ubiquitin-like protein ligase binding, protein serine/threonine kinase activity, ubiquitin protein ligase binding, serine hydrolase activity, protein tyrosine kinase activity. These results suggest that the candidate BA-LUAD overlapping targets are associated with xenobiotic and inflammatory responses, ROS metabolic processes, and kinase-related molecular functions, indicating possible involvement of redox regulation, signal transduction, and protein-modification processes in LUAD.

KEGG analysis of candidate targets identified 133 statistically enriched pathways (**Supplementary Table 5**), of which the top 20 are visualized in **Figure 4B**. Notably, several LUAD-related pathways were highlighted, including PI3K-AKT signaling pathway, MAPK signaling pathway, Chemical carcinogenesis-ROS, Proteoglycans in cancer, Chemical carcinogenesis-receptor activation, Th17 cell differentiation, Apoptosis, IL-17 signaling pathway, Endocrine resistance, and the p53 signaling pathway. To provide a more intuitive pathway-level view, the PI3K-AKT signaling pathway was further visualized and is provided in **Supplementary Figure 1**. Additionally, the top 50 enriched KEGG pathways were categorized into four major functional groups: Environmental Information Processing, Cellular Processes, Organismal Systems, and Human Diseases (**Supplementary Figure 2**). These results suggest that the candidate BA-LUAD target network is enriched in pathways related to oncogenic signaling (such as PI3K-AKT, MAPK, and p53), the tumor immune microenvironment (such as Th17 cell differentiation) (20), and ROS-related chemical carcinogenesis.

Among the three protein clusters identified in Section 3.3, Cluster 1 showed the highest connectivity score (7.524), whereas Clusters 2 and 3 showed lower-density network structures. Therefore, subsequent KEGG enrichment analysis was performed on Cluster 1 to examine whether pathways identified in the global enrichment analysis were also represented within this more densely connected subnetwork. The results revealed that genes within Cluster 1 were significantly enriched in Chemical carcinogenesis - receptor activation, Proteoglycans in cancer, PI3K-AKT signaling pathway, Apoptosis, and p53 signaling pathway (**Figure 4C**), all of which were also among the top pathways identified in the global KEGG analysis. These findings suggest that the major enriched pathways identified in the global KEGG analysis were also represented within the most cohesive PPI subnetwork, supporting their prioritization as candidate biological contexts for further investigation. In addition, Cluster 1 was enriched in the estrogen signaling pathway and endocrine resistance, suggesting that ESR1-related hormone-response signaling may represent another candidate context within the BA-LUAD network.

### 3.5 Construction of target-GO-BP/KEGG network

Among the 117 targets, 57 were linked to the top 5 enriched GO-BP terms, with response to xenobiotic stimulus exhibiting the highest connectivity (**Figure 5A**). Concurrently, 39 targets were enriched in the top 5 LUAD-associated KEGG pathways, with PI3K-AKT signaling pathway and MAPK signaling pathway demonstrating the highest target connectivity, followed by Chemical carcinogenesis - reactive oxygen species (**Figure 5B**).

**Figure 5:**
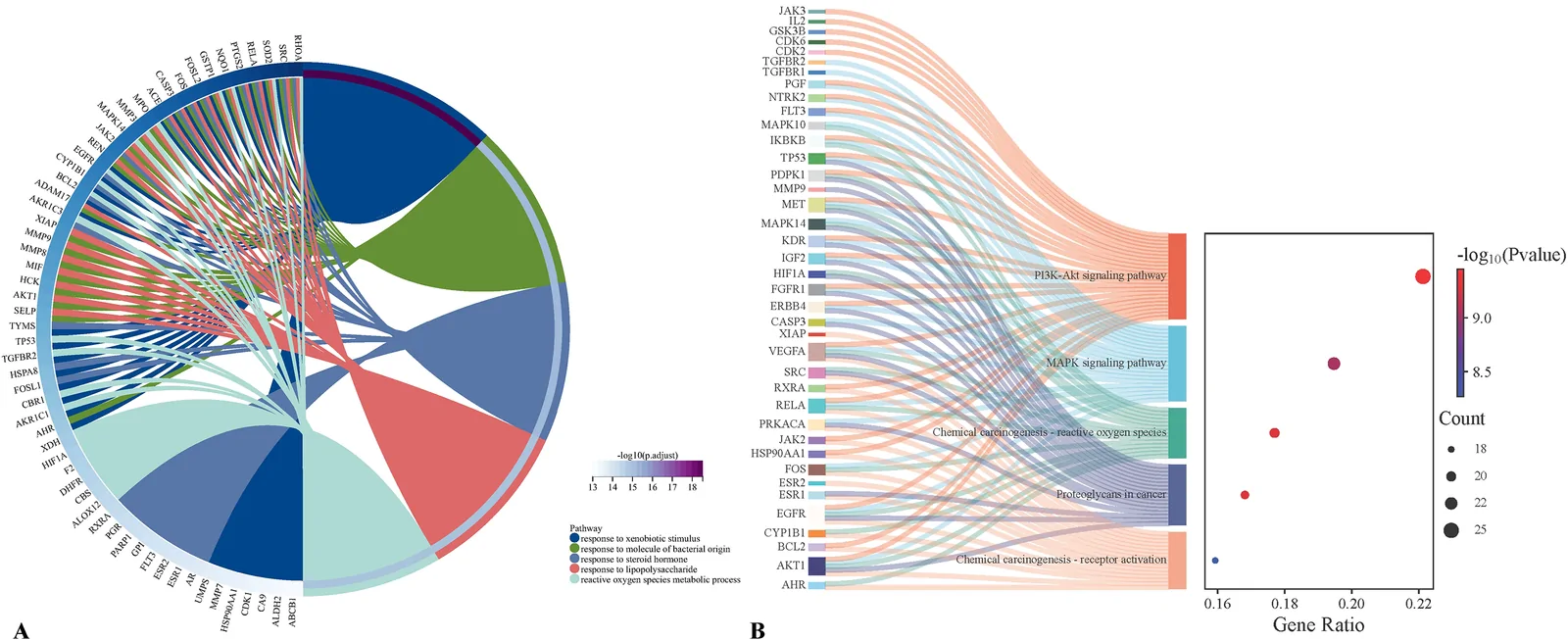
Construction of target-GO-BP/KEGG network. **(A)** Chord diagram of target-GO-BP. **(B)** Sankey bubble plot of target-KEGG.

Subsequently, a total of 46 target genes were identified to participate in both the key GO-BP terms and LUAD-related KEGG pathways. Detailed enrichment information for these targets is provided in **Supplementary Table 6**, and their distribution across pathways is visualized in the Sankey diagram in **Supplementary Figure 3**. The top 16 target genes are listed in **Table 2**, among which FOS and RELA exhibited the highest occurrence rates. It is noteworthy that among the six hub genes identified in the PPI network, except for ESR1, which ranked 23rd in total frequency and was involved in 2 BP terms and 3 KEGG pathways, AKT1, EGFR, BCL2, TP53, and HIF1A ranked highly in this analysis. This suggests that these targets were recurrently represented across both BP and KEGG enrichment results, supporting their prioritization as candidate nodes within the BA-LUAD overlapping target network for further validation.

**Table 2:** The top 16 targets of Target-GO-BP/KEGG network.

Gene	Total (n)	GO-BP (n)	KEGG (n)	Proportion (%) ^*^
FOS	14	7	7	70
RELA	14	7	7	70
**AKT1**	13	6	7	65
**EGFR**	13	7	6	65
**BCL2**	12	7	5	60
SRC	12	8	4	60
CASP3	11	6	5	55
**TP53**	11	5	6	55
MAPK14	10	4	6	50
CYP1B1	9	7	2	45
JAK2	9	6	3	45
SOD2	9	8	1	45
**HIF1A**	8	5	3	40
NQO1	7	6	1	35
PTGS2	7	6	1	35
VEGFA	7	2	5	35

^*^ Proportion was defined as (occurrence frequency**/**total number of terms) × 100%. Bold gene names indicate candidate hub genes identified in the PPI network.

### 3.6 Immune infiltration analysis

First, the estimated relative fractions of 22 immune cell types in TCGA-LUAD bulk RNA-seq samples were obtained using CIBERSORT (**Supplementary Figure 4**). Subsequently, differences in the estimated immune-cell fractions between LUAD and normal lung tissues were analyzed (**Supplementary Figure 5**). The estimated fractions of plasma cells, activated memory CD4 T cells, follicular helper T cells, regulatory T cells (Tregs), M1 macrophages, and resting dendritic cells were significantly higher in LUAD tissues than in normal tissues (nominal *p* < 0.001). In contrast, the estimated fractions of resting memory CD4 T cells, resting NK cells, monocytes, M0 and M2 macrophages, resting mast cells, eosinophils, and neutrophils were significantly lower in LUAD tissues (nominal *p* < 0.001).

In addition, the correlations between the expression levels of the six hub genes and the infiltration levels of various immune cell types were assessed and visualized in the form of a heatmap (**Figure 6A**). Plasma cells showed a negative correlation with all hub genes, with nominally significant associations observed for AKT1, EGFR, ESR1, and HIF1A. Resting memory CD4 T cells exhibited a positive correlation with all six genes, among which BCL2, EGFR, and ESR1 showed nominally significant associations. Notably, among the six candidate hub genes, HIF1A showed the broadest pattern of nominally significant associations with the estimated immune-cell fractions, including positive correlations with neutrophils and activated memory CD4 T cells, and negative associations with memory B cells, activated NK cells, plasma cells, follicular helper T cells, and Tregs.

**Figure 6:**
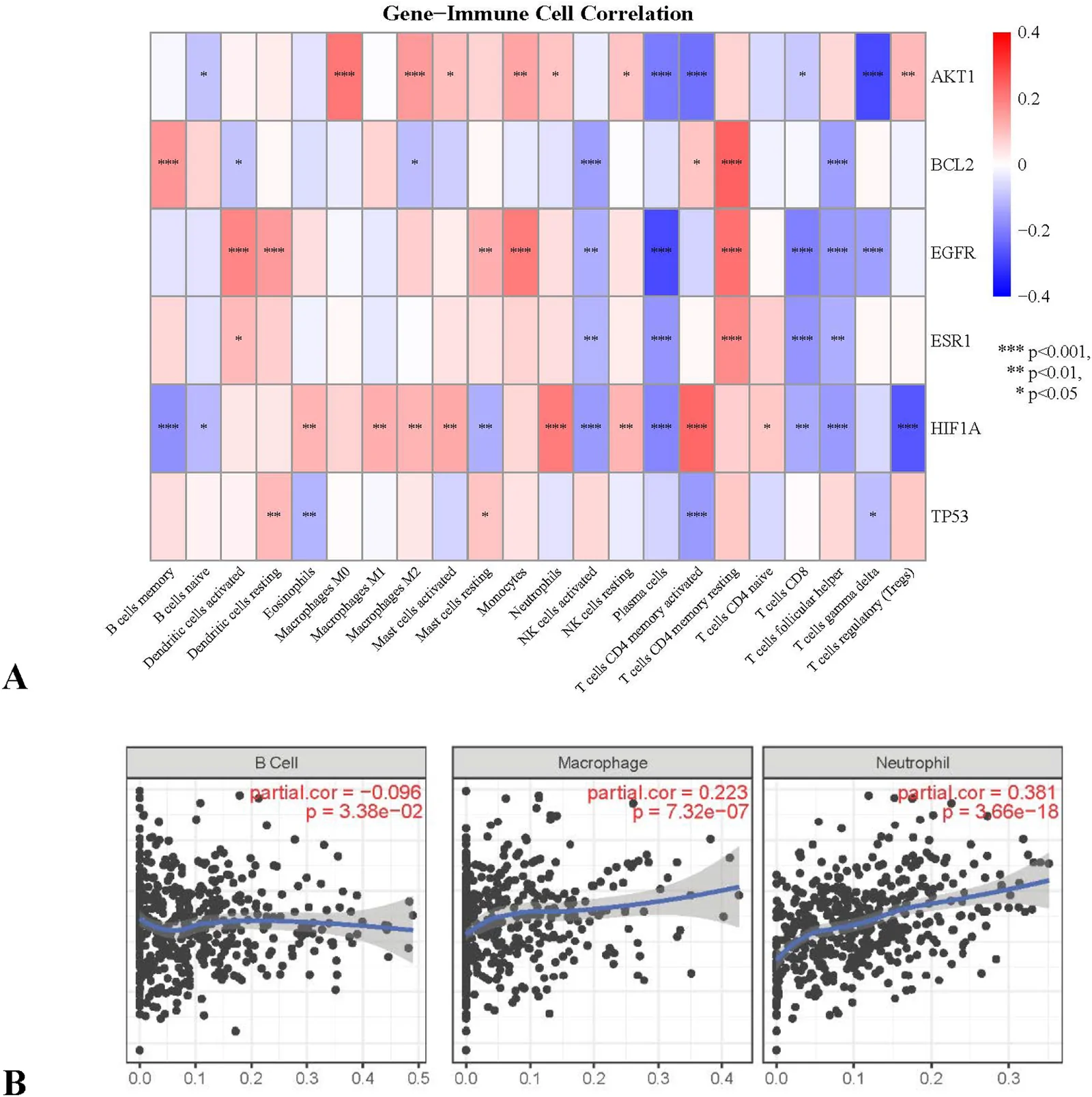
Immune infiltration analysis. **(A)** Correlation heatmap between six hub genes and 22 immune cell types estimated by CIBERSORT. Asterisks indicate nominal p-values: *p < 0.05; **p < 0.01; ***p < 0.001. **(B)** TIMER-based associations of HIF1A expression with B-cell, macrophage, and neutrophil infiltration.

To provide a complementary computational cross-check, TIMER analysis was further performed. Partially consistent with the CIBERSORT results, HIF1A showed a positive association with neutrophil infiltration and macrophage infiltration, and a weak negative association with B-cell infiltration (**Figure 6B**). These results provide complementary computational support for HIF1A-related immune-infiltration associations, particularly those involving neutrophil- and macrophage-related estimates. The discrepancy in CD8+ T cells and the non-significant total CD4+ T cells result may reflect differences in immune-cell classification between CIBERSORT and TIMER, as CIBERSORT distinguishes multiple T-cell subsets whereas TIMER reports broader immune-cell categories. The complete TIMER results are provided in **Supplementary Figure 6**. Because both approaches are based on computational deconvolution of bulk RNA-seq data, their partial concordance should be interpreted as a cross-method computational comparison rather than independent validation.

### 3.7 Molecular docking analysis

Molecular docking was used to assess the computational plausibility of BA poses within the selected structures of the six hub proteins. Binding affinity was used as a computational estimate of ligand-receptor compatibility, with lower values indicating more favorable predicted binding. Generally, a binding affinity below -4.25 kcal/mol, -5.0 kcal/mol, or -7.0 kcal/mol has been used as an empirical reference suggesting potential binding activity, relatively favorable binding activity, or strong predicted binding affinity, respectively (21). After five repeated docking runs, the median binding affinities of BA with AKT1, BCL2, EGFR, ESR1, HIF1A, and TP53 were -9.619, -7.864, -8.158, -8.749, -7.576, and -7.643 kcal/mol, respectively. The docking conformations are shown in **Figures 7A–F**. According to the above empirical criteria, these values suggest potentially favorable predicted docking compatibility, but do not confirm direct binding, target specificity, or functional regulation. Positive-control docking was performed to provide reference values for interpreting BA docking scores. The reference compounds showed favorable docking affinities, including MK-2206 with AKT1 (-10.7 kcal/mol), ABT-199 with BCL2 (-10.55 kcal/mol), gefitinib with EGFR (-8.101 kcal/mol), tamoxifen with ESR1 (-9.702 kcal/mol), and YC-1 with HIF1A (-7.366 kcal/mol) (**Supplementary Figure 7**). Compared with the corresponding positive controls, BA showed comparable predicted docking scores for several hub proteins, providing additional computational support for the plausibility of the docking results. In addition, the RMSD values between the redocked ligand poses and the original crystallographic ligand poses were 1.746 Å for AKT1, 1.868 Å for EGFR, 1.779 Å for ESR1, and 0.894 Å for HIF1A. RMSD values below 2 Å supported the ability of the selected docking protocol to reproduce the corresponding crystallographic poses, thereby improving confidence in the technical consistency of the docking workflow.

**Figure 7:**
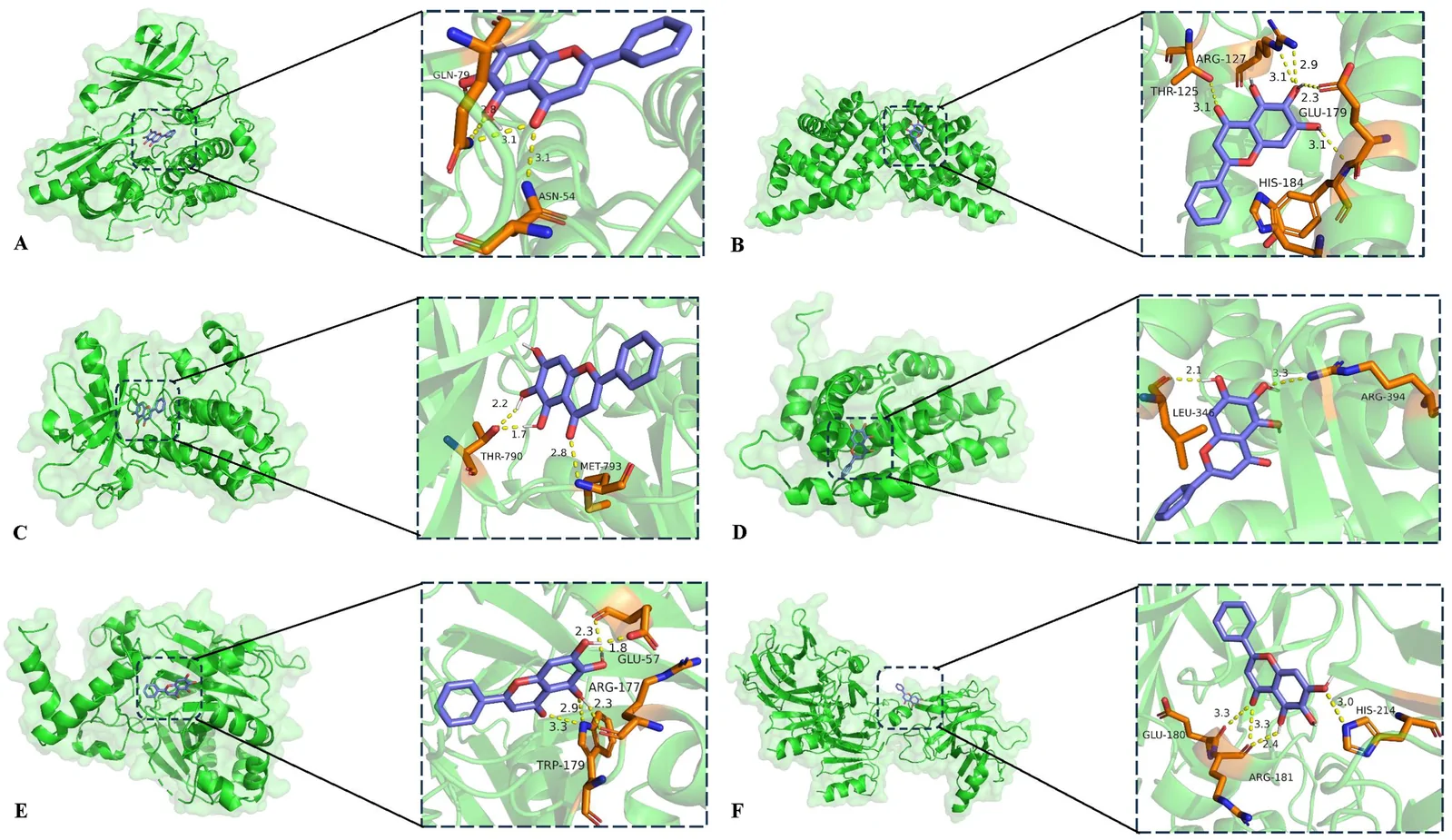
Molecular docking results of hub targets and BA. **(A)** AKT1 (score: -9.619). **(B)** BCL2 (score: -7.864). **(C)** EGFR (score: -8.158). **(D)** ESR1 (score: -8.749). **(E)** HIF1A (score: -7.576). **(F)** TP53 (score: -7.643).

### 3.8 Expression and prognostic analysis of hub genes

To further assess the expression and prognostic associations of the hub targets in LUAD, this study integrated multi-dimensional bioinformatics data. First, protein expression profiles from HPA and transcriptomic data from the GEPIA2 database were used to evaluate the expression characteristics of the hub genes in LUAD and normal lung tissues. As shown in **Supplementary Figure 8** and **Figure 8A**, the expression level of TP53 was significantly higher in LUAD compared to normal lung tissue at both the protein and transcriptomic levels. In contrast, AKT1, EGFR, ESR1, and HIF1A exhibited lower expression in LUAD than in normal tissue. Notably, BCL2 showed no detectable protein staining in either LUAD or normal lung tissue according to the HPA database. In the representative LUAD sample, tumor cells were annotated as “Not detected,” with negative staining intensity and no positive-cell quantity; similarly, alveolar cells and macrophages in the representative normal lung sample were annotated as “Not detected,” with negative intensity and no positive-cell quantity. In contrast, BCL2 displayed significantly lower mRNA expression in LUAD based on transcriptomic analysis. **Figure 8B** and **Supplementary Figure 8** further illustrate the association between hub gene expression levels and tumor stages in LUAD. Among the six hub targets, only BCL2 showed statistically significant expression variation across pathological stages (*F* = 2.73, *p* = 0.0434), with lower expression observed in the later-stage groups. Moreover, survival analysis (**Figure 8C**) revealed that high expression of BCL2 was significantly associated with better OS (*HR* = 0.73, *p* = 0.04), whereas high expression of HIF1A was associated with poorer prognosis (*HR* = 1.4, *p* = 0.035).

**Figure 8:**
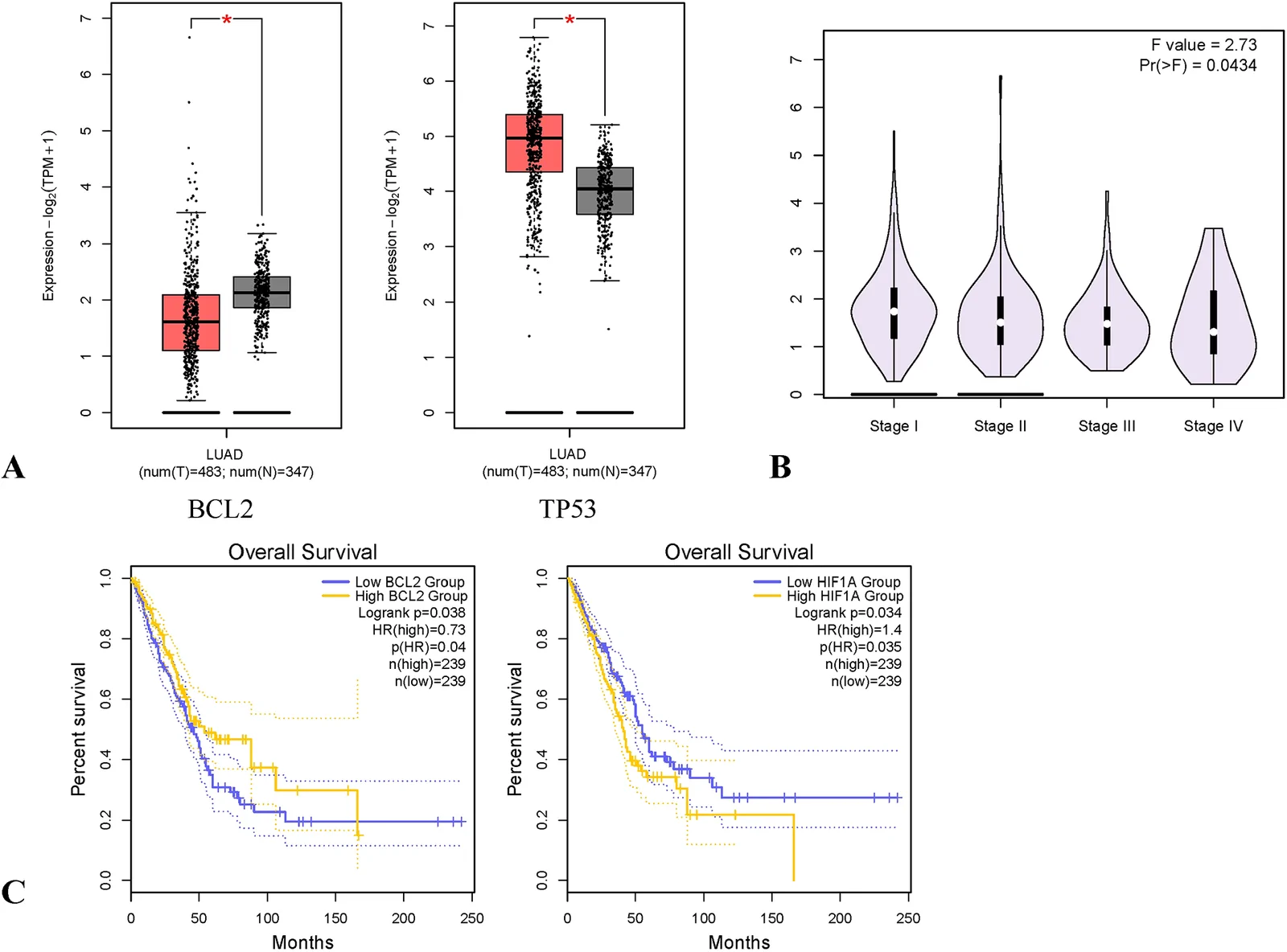
Expression and prognostic analysis of hub genes. **(A)** The expression of BCL2 and TP53 at the transcriptome levels in normal versus LUAD samples. LUAD samples, n = 483; normal samples, n = 347; *p < 0.05. **(B)** The expression of BCL2 at different stages of LUAD. **(C)** Survival analysis of BCL2 and HIF1A expression and LUAD prognosis. High-expression group, n = 239; low-expression group, n = 239. HR = hazard ratio.

### 3.9 Cell viability assay

A CCK-8 assay was performed to evaluate the effect of BA on A549 cell viability. BA reduced A549 cell viability in a concentration-dependent manner at both 24 h and 48 h (**Figure 9**). The estimated IC_50_ values were 54.93 μM at 24 h and 22.83 μM at 48 h, indicating that BA reduced A549 cell viability *in vitro*.

**Figure 9:**
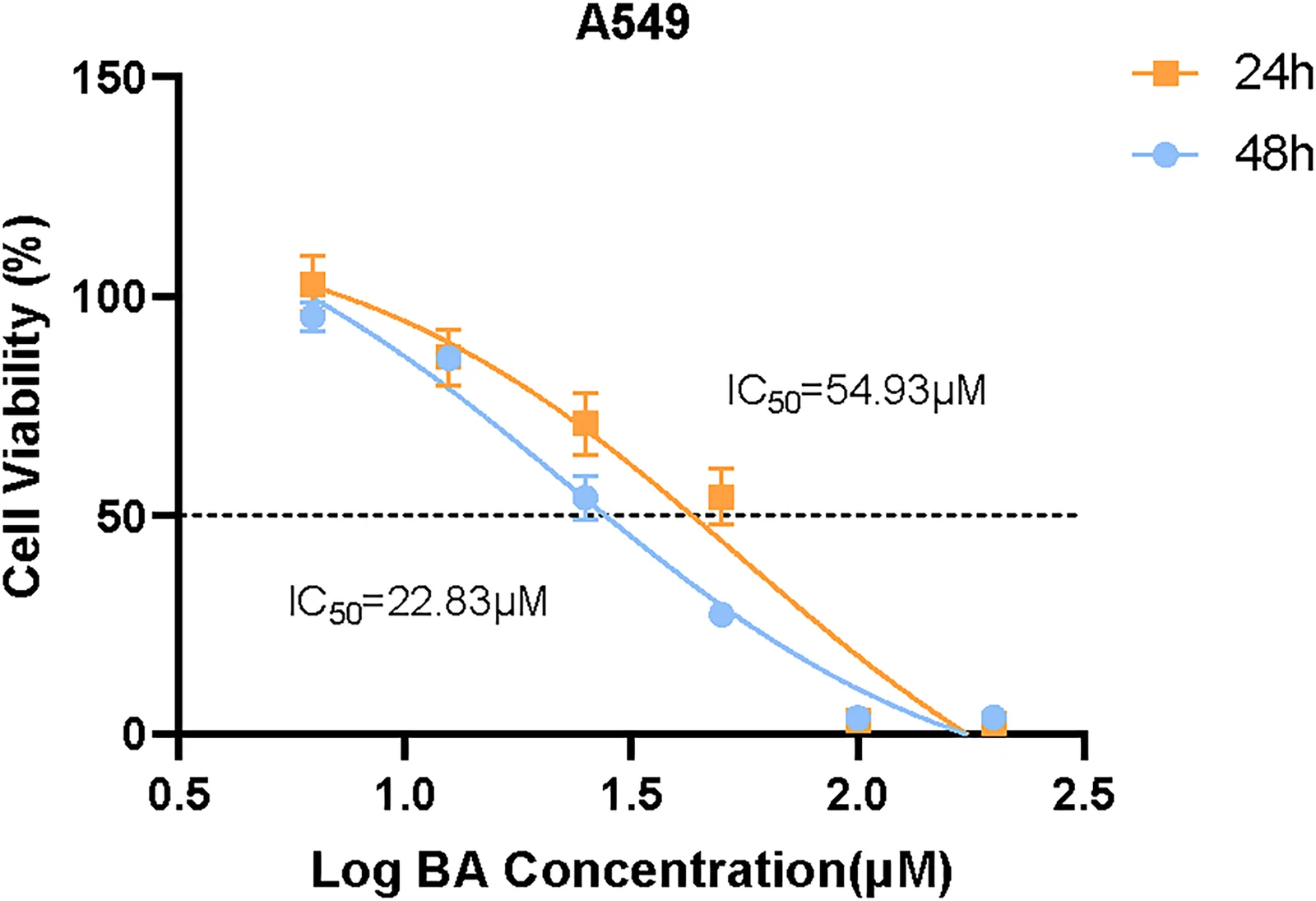
CCK-8 analysis of cell viability after BA treatment. Data are presented as mean ± SD from three independent biological experiments (n = 3), with three technical replicate wells per condition in each experiment. Error bars represent SD. IC_50_ values were estimated using a log(inhibitor) versus response three-parameter nonlinear regression model. BA, baicalein; IC_50_, half maximal inhibitory concentration.

### 3.10 Western blot

Considering the above computational analyses, in which AKT1 was recurrently represented across enriched pathways and was closely associated with the PI3K-AKT signaling context, we further performed western blot analysis to provide preliminary experimental support for AKT1-related signaling involvement. As shown in **Figure 10A** and **Supplementary Figure 9**, BA treatment led to a concentration-dependent reduction in p-AKT1 levels, whereas the expression of total AKT1 remained largely unchanged compared with the control group. A downward trend was observed at 12.5 μM but did not reach statistical significance, whereas p-AKT1 was significantly reduced at 25 μM (*p* < 0.05) and further decreased at 50 μM (*p* < 0.001) (**Figure 10B**). These findings are consistent with reduced AKT1 phosphorylation under the tested conditions, while total AKT1 protein abundance remained largely unchanged. In addition, because apoptosis-related pathways were identified in the KEGG enrichment analysis and BCL2 was prioritized as a candidate hub gene within this computational context, BCL2 protein expression was also examined. As shown in **Figures 10C, D**, BA treatment reduced BCL2 protein levels in A549 cells in a dose-dependent manner, with the most pronounced decrease observed at 50 μM (*p* < 0.001). These results demonstrate a BA-associated reduction in BCL2 protein abundance in A549 cells under the tested conditions.

**Figure 10:**
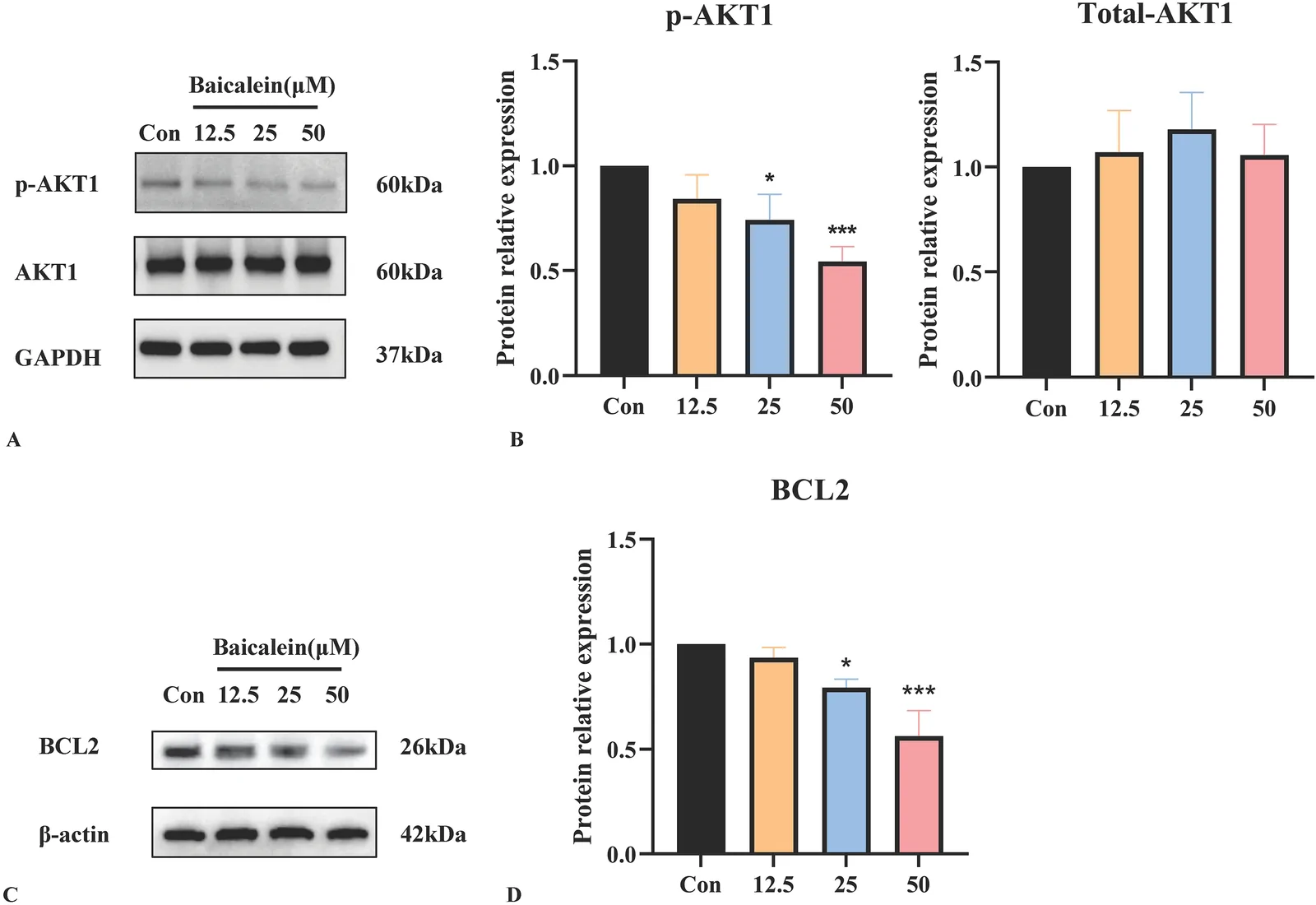
Western blot analysis. **(A)** Representative western blot images showing the protein expression levels of p-AKT1, total AKT1, and GAPDH in A549 cells treated with BA (12.5, 25, and 50 μM). **(B)** Densitometric analysis of p-AKT1 and total AKT1 protein expression levels relative to GAPDH. **(C)** Representative western blot images showing the protein expression levels of BCL2 in A549 cells treated with BA. **(D)** Densitometric analysis of BCL2 protein expression levels relative to β-actin. Quantitative data are presented as mean ± SD from three independent biological experiments (n = 3), and error bars represent SD. Con, control; p-AKT1, phospho-AKT1. *p < 0.05, ***p < 0.001.

## 4 Discussion

*Scutellaria baicalensis* Georgi is a perennial botanical drug belonging to the Lamiaceae family, first documented in the classical Chinese pharmacopeia Shennong Bencao Jing, written between 200 and 300 AD. It has been widely used in TCM prescriptions and is regarded as a versatile medicinal plant (9). Modern pharmacological research has demonstrated that the diverse biological activities of *Scutellaria baicalensis* Georgi are closely associated with its flavonoid metabolites. BA, as one of its primary active metabolites, exhibits remarkable potential in anti-inflammatory, antioxidant, and antitumor applications (3). Whether derived from whole botanical drugs or isolated flavonoid metabolites, TCM-derived interventions are often proposed to act through multiple targets and diverse mechanisms (13, 22). The distinctive contribution of this study is the LUAD-specific evaluation of these candidates across transcriptomic alterations, co-expression modules, clinical outcomes, immune-cell estimates, structural compatibility, and focused experimental readouts. In particular, the convergence of HIF1A-related prognostic and immune associations, together with the context-dependent patterns of BCL2 across tumor stage, survival, and BA treatment, provides testable observations beyond conventional target-overlap and enrichment analyses. These findings refine the biological context and experimental priority of established BA-related candidates, although their mechanistic roles require further functional validation.

In this study, CCK-8 assays showed that BA reduced A549 cell viability in a concentration- and time-dependent manner, with a lower IC_50_ at 48 h than at 24 h. Integrated analysis of drug-related databases, disease-associated databases, TCGA-LUAD differential expression, WGCNA, and PPI network topology prioritized six candidate hub genes: EGFR, AKT1, TP53, BCL2, HIF1A, and ESR1. Among these candidates, AKT1 and BCL2 were selected for focused experimental assessment because both were recurrently represented across the integrated analyses and represented two prominent biological contexts within the candidate network. This focused selection was intended to provide preliminary experimental assessment of representative nodes. Clinical database analyses further identified stage- and prognosis-related features of BCL2 and an adverse prognostic association of HIF1A. Molecular docking generated favorable predicted scores for the selected proteins, while positive-control docking and redocking supported the technical consistency of the docking workflow. These docking results provide preliminary structural indications of potential ligand-protein compatibility and do not establish ligand specificity, direct target engagement, or functional modulation. Although the positive-control docking and redocking analyses provide pharmacological context and support the technical consistency of the docking workflow, they do not establish the dynamic stability of the predicted BA-protein complexes. Molecular dynamics simulations were not performed in the present study but could provide additional structural validation.

As noted, the PI3K/AKT pathway is critical to various cellular processes and represents a principal signaling axis in cancer development (23). The PI3K/AKT pathway showed high target connectivity in the enrichment analysis and was also represented within the most densely connected PPI cluster. AKT1, a central effector of this pathway, is activated through phosphorylation and participates in the regulation of tumor-cell survival, metabolism, apoptosis resistance, and therapeutic response in NSCLC (24, 25). In the present study, AKT1 was recurrently represented across enriched pathways and yielded the most favorable predicted docking score among the six candidate proteins. Western blot analysis further showed that BA treatment reduced AKT1 phosphorylation, while total AKT1 protein abundance remained largely unchanged. These findings suggest that AKT1-associated signal transduction may represent an important component of the BA response in A549 cells.

BCL2, a major anti-apoptotic member of the BCL2 protein family, was also recurrently represented in apoptosis- and PI3K/AKT-related pathways, and its protein expression decreased following BA treatment (26). Previous studies provide a biological basis for considering a potential relationship between AKT-related signaling and BCL2-associated survival regulation in the BA context. Yu et al. reported that BA suppressed PI3K/Akt/NF-κB signaling and reduced NF-κB-mediated anti-apoptotic activity in cisplatin-resistant A549 cells (27). Lu et al. further showed that BA reduced PI3K and p-Akt levels through the miR-424-3p/PTEN pathway and promoted apoptosis in A549 and H460 cells (28). In parallel, Kiartivich et al. found that BA treatment decreased BCL2 expression, increased Bax and caspase-3 expression, and enhanced apoptosis-related responses in A549 cells (29). In the present study, BA treatment was associated with concurrent reductions in p-AKT1 and BCL2 protein expression in A549 cells. However, these parallel observations do not establish a causal or upstream-downstream relationship between AKT1 and BCL2. The observed changes may reflect a shared response to BA treatment, independent regulation through distinct pathways, or an indirect relationship that cannot be resolved by the current data. Therefore, an AKT1-BCL2 connection should be regarded only as a working hypothesis for future investigation. Genetic or pharmacological perturbation of AKT1 and BCL2, followed by functional rescue experiments, will be required to test this hypothesis directly. It should also be emphasized that the apoptosis-related context in the present study was primarily derived from pathway-enrichment analysis and the known biological function of BCL2. Although BA treatment reduced BCL2 protein abundance, the present experiments did not determine whether this reduction resulted from transcriptional regulation, altered protein stability or degradation, or other post-transcriptional mechanisms.

Importantly, the apparently paradoxical BCL2 pattern observed in the present study may provide additional biological context rather than simply representing an inconsistency. Although BCL2 is classically regarded as a pro-survival anti-apoptotic protein, its prognostic relevance is context-dependent and has been reported to vary across different cancer settings (30). In NSCLC, previous studies have shown that higher BCL2 protein expression is associated with improved survival, suggesting that BCL2 does not uniformly behave as an adverse oncogenic marker in lung cancer, with this favorable prognostic association appearing more pronounced in non-squamous tumors, including LUAD, than in squamous cell carcinoma (31, 32). Thus, the favorable survival association observed in the present analysis is consistent with prior clinical evidence rather than being uniquely contradictory to the canonical anti-apoptotic function of BCL2. In LUAD specifically, accumulating evidence indicates that tumor-cell survival may be sustained by alternative anti-apoptotic dependencies, particularly BCL2L1 and MCL1, while BCL2 expression tends to be relatively low in many LUAD models and tissues (33, 34). This concept is further supported by functional studies showing that MCL1 is frequently gained and can represent an important survival dependency in LUAD, while KRAS-mutant NSCLC models may show marked sensitivity to combined targeting of MEK and MCL1 (35, 36). These findings suggest that a relatively low level of BCL2 does not imply loss of anti-apoptotic dependence altogether, but may instead reflect redistribution of survival dependence toward other BCL2-family proteins. This background helps explain why reduced BCL2 mRNA expression in LUAD, its further decrease with advancing tumor stage, and the favorable prognostic association of higher BCL2 expression can coexist in the present study. The HPA findings should also be interpreted within this context. In the representative LUAD specimen, tumor cells were annotated as “not detected,” with negative staining intensity and no positively scored tumor cells; similarly, alveolar cells and macrophages in the representative normal lung specimen were annotated as “not detected,” with negative intensity and no positively scored cells. However, a “not detected” HPA annotation indicates that no clear immunohistochemical signal was observed in the examined tissue sections under the corresponding assay conditions and should not be interpreted as definitive absence of BCL2 protein across all LUAD tissues. In addition, transcript abundance is not always proportional to detectable protein abundance because protein levels can be influenced by post-transcriptional regulation, translational efficiency, protein turnover, assay sensitivity, and tissue heterogeneity (37). Taken together, the lower BCL2 expression, favorable prognostic association, and BA-associated reduction in BCL2 protein are not necessarily mutually contradictory. Rather, they highlight the context-dependent position of BCL2 within a broader anti-apoptotic network in LUAD and suggest that BCL2 should not be interpreted as a uniformly dominant survival factor in this disease.

HIF1A was prioritized primarily through its clinical and immune-infiltration associations. Higher HIF1A expression was associated with poorer OS, consistent with previous LUAD-focused evidence linking elevated HIF1A expression to adverse clinical outcomes (38). HIF1A is a major regulator of hypoxic adaptation and may influence tumor metabolism, survival, and immune-related transcriptional states within the TME (39, 40). The enrichment of hypoxia- and ROS-related processes in the candidate network provides additional biological context for considering HIF1A in BA-associated LUAD hypotheses. Previous studies have also reported that BA can affect HIF1A accumulation and activity in other experimental settings through ROS- and PI3K/Akt-related processes (41, 42). Direct evidence for BA-mediated HIF1A regulation in LUAD remains limited.

CIBERSORT analysis showed that HIF1A expression was associated with multiple computationally estimated immune-cell fractions. TIMER provided a complementary computational comparison and showed partially concordant associations, particularly positive associations with neutrophil- and macrophage-related infiltration and a negative association with B-cell infiltration. Differences in the CD8^+^ and CD4^+^ T-cell results may reflect variations in immune-cell definitions and deconvolution algorithms between the two methods. These findings suggest that HIF1A expression may be linked to hypoxia-associated immune states in the LUAD microenvironment (9). As a central transcription factor involved in hypoxic adaptation, HIF1A has been shown to facilitate Th17 polarization under hypoxic conditions through multiple synergistic mechanisms, thereby contributing to immune-state heterogeneity within the TME (27, 43). However, these associations should be interpreted as transcriptomic correlations inferred from bulk RNA-seq data, rather than evidence that BA directly regulates immune-cell abundance or function. Validation using single-cell, spatial, or immune-cell-specific approaches would strengthen the biological interpretation of these associations.

EGFR, TP53, and ESR1 provide additional candidate contexts within the BA-LUAD network. EGFR may connect the candidate network with receptor-tyrosine-kinase and PI3K/AKT-related signaling, consistent with previous evidence of BA-associated effects in EGFR-related cancer models (44, 45). TP53 was associated with p53-related stress-response pathways, and previous studies have linked BA with p53-associated apoptotic responses in A549 cells and experimental lung carcinogenesis (46). ESR1 was represented in steroid-hormone response, estrogen signaling, and endocrine-resistance pathways, suggesting a possible hormone-response context (47–49). Experimental assessment of EGFR, TP53, and ESR1 was not performed in the present study, and their roles remain exploratory candidate hypotheses. Likewise, the current experimental observations do not establish that AKT1 and BCL2 account for the full anti-proliferative effect of BA. Other prioritized candidates, including EGFR, TP53, HIF1A, and ESR1, as well as additional pathways represented in the candidate network, may contribute independently or cooperatively to the overall cellular response.

Taken together, the enrichment results, focused experimental findings, and immune-infiltration analyses support a multi-target working framework in which AKT1-associated signaling and BCL2-associated cell-survival regulation represent two experimentally examined components, while HIF1A provides a complementary prognostic and immune-microenvironmental context. The concurrent decreases in AKT1 phosphorylation and BCL2 protein expression identify these proteins as two candidate components associated with the cellular response to BA; however, the present data do not establish whether these changes are causally connected. HIF1A contributes a complementary prognostic and immune-microenvironmental context based on clinical and bulk RNA-seq-derived associations. AKT1-associated signaling, BCL2-associated cell-survival regulation, and HIF1A-associated immune patterns should therefore be considered complementary research directions within the broader BA-LUAD candidate network.

From a pharmacological perspective, interpretation of the BA-associated candidate mechanisms should also consider exposure, bioavailability, and formulation-related factors. Although BA has shown experimental activity in lung cancer models, its translational application may be constrained by pharmacokinetic limitations, including poor aqueous solubility, extensive metabolism, and relatively limited oral bioavailability (50). Pharmacokinetic studies have shown that BA is absorbed and extensively glucuronidated, while its reported oral bioavailability varies across experimental models and formulations (51). Future studies should therefore evaluate BA-related mechanisms under pharmacologically relevant exposure conditions. Emerging delivery strategies, such as nanocarrier-based delivery systems, may help improve pharmacokinetic behavior, enhance tumor targeting, and support combination therapy strategies, offering a practical direction for future BA-based translational research in LUAD (52).

Despite the integrative and multi-layered design of this study, several limitations warrant careful consideration. First, a substantial proportion of the findings was derived from public target-prediction and disease-association databases, TCGA transcriptomics, WGCNA, network topology, enrichment analysis, molecular docking, and clinical database analyses. These methods are valuable for candidate prioritization, but their results may be affected by database coverage, literature bias, computational assumptions, and the recurrent selection of highly connected cancer-related genes (53). The 117 overlapping targets and six hub genes therefore constitute a prioritized candidate network that requires further biological evaluation. Accordingly, the multi-layered prioritization strategy reduces reliance on any single target-prediction source but cannot establish the specificity of BA for the prioritized candidates. In addition, module preservation was not formally evaluated; therefore, the reproducibility of the WGCNA-derived modules across independent datasets remains to be established. Meanwhile, nonspecific interactions or assay interference associated with the polyphenolic structure of BA cannot be fully excluded (54). A further important limitation is that only AKT1 and BCL2 were selected for focused experimental assessment among the prioritized candidate targets. Therefore, the current data cannot determine the relative contribution of AKT1, BCL2, or other predicted targets to the overall pharmacological effect of BA. The observed changes in AKT1 phosphorylation and BCL2 protein expression support the involvement of these two candidates in the A549 cellular response, but the present data do not establish their relative contribution to the overall multi-target activity of BA. Genetic perturbation approaches, such as siRNA-mediated knockdown or CRISPR/Cas9-based manipulation, could help quantify the target-specific contribution of individual candidates. Second, the cell-based assessment was conducted only in A549 cells. The CCK-8 and western blot results support BA-associated changes in cell viability, AKT1 phosphorylation, and BCL2 protein expression under the tested conditions, but the generalizability of these observations across different LUAD molecular backgrounds remains uncertain. Moreover, the experimental analyses were based on three independent biological experiments, which limits statistical precision and the sensitivity of formal assumption testing. Evaluation in additional LUAD cell lines with distinct molecular backgrounds, together with genetic or pharmacological perturbation of AKT1 and BCL2, will be required to assess the generalizability of these findings and determine whether the observed changes in AKT1 phosphorylation and BCL2 expression are functionally connected. In addition, apoptosis was not directly quantified, and the mechanism underlying the observed reduction in BCL2 protein abundance was not determined; apoptosis-specific assays and analyses of BCL2 regulation will therefore be required in future studies. Third, CIBERSORT and TIMER are both based on computational deconvolution of bulk RNA-seq data. These complementary analyses identified HIF1A-related immune-infiltration associations but cannot resolve cell-type-specific transcriptional states or spatial interactions within the LUAD microenvironment. Public single-cell RNA-seq datasets, spatial-transcriptomic data, and immune-cell-specific experiments would provide higher-resolution validation. Finally, the present study did not include *in vivo* experiments. Appropriate animal models will be required to assess whether the candidate molecular and immune-related patterns observed in the current analyses are maintained under *in vivo* exposure and tumor-microenvironmental conditions. Overall, these limitations emphasize the hypothesis-generating nature of the proposed BA-LUAD network and define priorities for subsequent mechanistic and translational studies.

## 5 Conclusion

This study integrated network analysis, TCGA-LUAD transcriptomics, WGCNA, enrichment analysis, bulk RNA-seq-based immune-infiltration estimation, molecular docking, clinical database assessment, and focused cell-based experiments to prioritize BA-associated candidate mechanisms in LUAD. Computational analyses identified 117 candidate BA-LUAD overlapping targets and prioritized six hub genes, including EGFR, AKT1, TP53, BCL2, HIF1A, and ESR1. These candidates were mainly associated with PI3K-AKT/MAPK signaling, apoptosis-related regulation, ROS-related processes, p53 signaling, and immune-microenvironmental features. Molecular docking further suggested favorable predicted structural compatibility between BA and the six candidate proteins, while clinical database analyses highlighted the stage- and prognosis-related features of BCL2 and the adverse prognostic association of HIF1A. Additionally, HIF1A showed broad associations with computationally estimated immune-cell fractions. Experimentally, BA reduced A549 cell viability and decreased AKT1 phosphorylation and BCL2 protein expression. These findings identify AKT1-associated signaling and BCL2-associated cell-survival regulation as two experimentally observed candidate components of the BA response in A549 cells. However, the proposed BA-LUAD network remains hypothesis-generating and requires further validation in additional LUAD models, together with target-engagement, functional, and *in vivo* studies. Collectively, this study suggests that BA may have multi-pathway relevance in LUAD and provides a prioritized framework for future mechanistic validation and pharmacological optimization.

## Data Availability

The original contributions presented in the study are included in the article/**Supplementary Material**. Further inquiries can be directed to the corresponding author.
