# Supplementary material for: Exploring the mechanisms of baicalein in lung adenocarcinoma: an integrative preliminary study with focused experimental assessment in A549 cells
Source: Front Oncol. 2026 Oct 6;16:1912108. doi: 10.3389/fonc.2026.1912108 (PMC13680175; doi:10.3389/fonc.2026.1912108)
Supplement: Supplementary file 1 [file DataSheet1.zip › Supplementary/Supplementary_Material.pdf]

Supplementary Figures

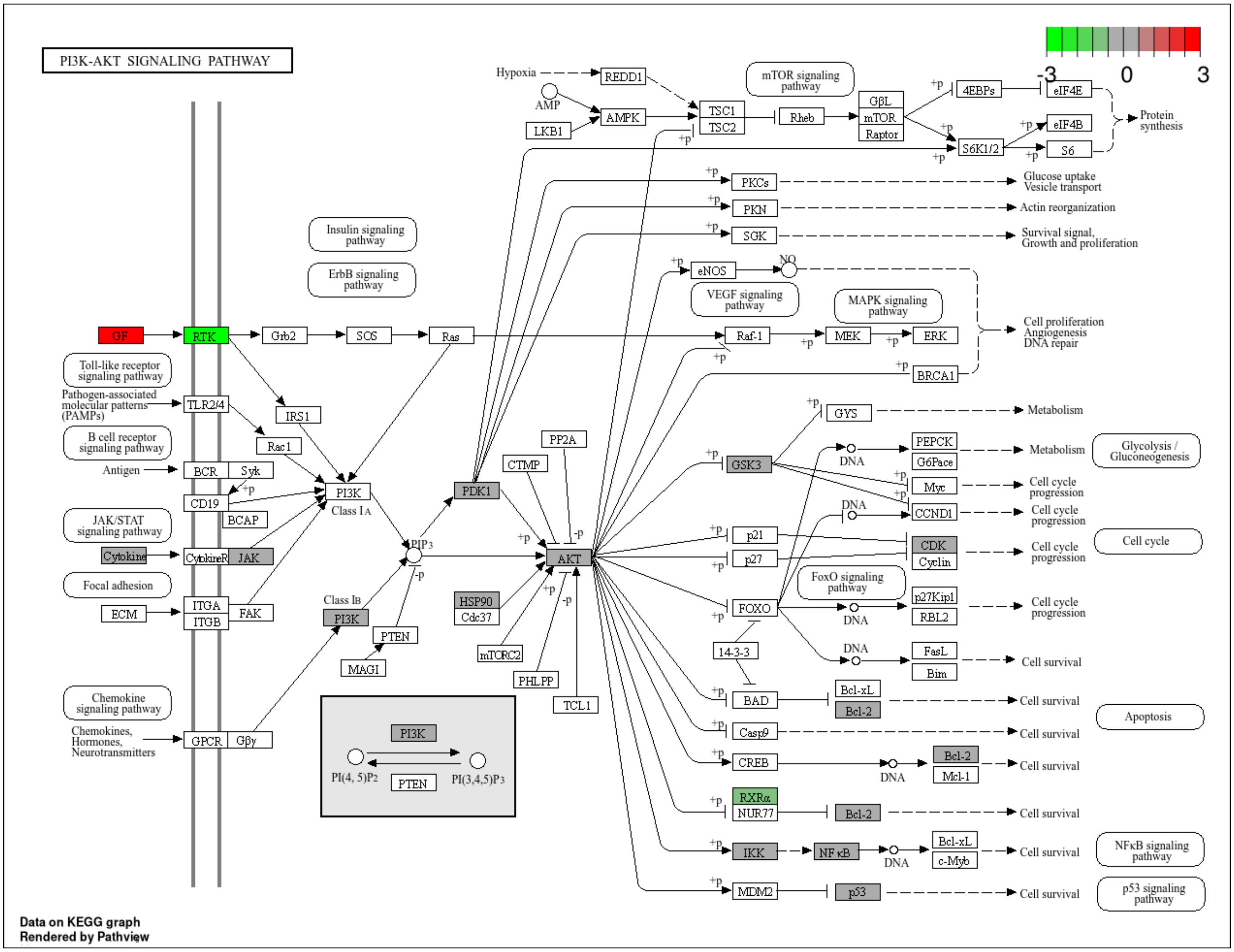

Supplementary Figure 1. The PI3K-AKT signaling pathway diagram. Red fill indicates upregulated genes; green fill indicates downregulated genes.

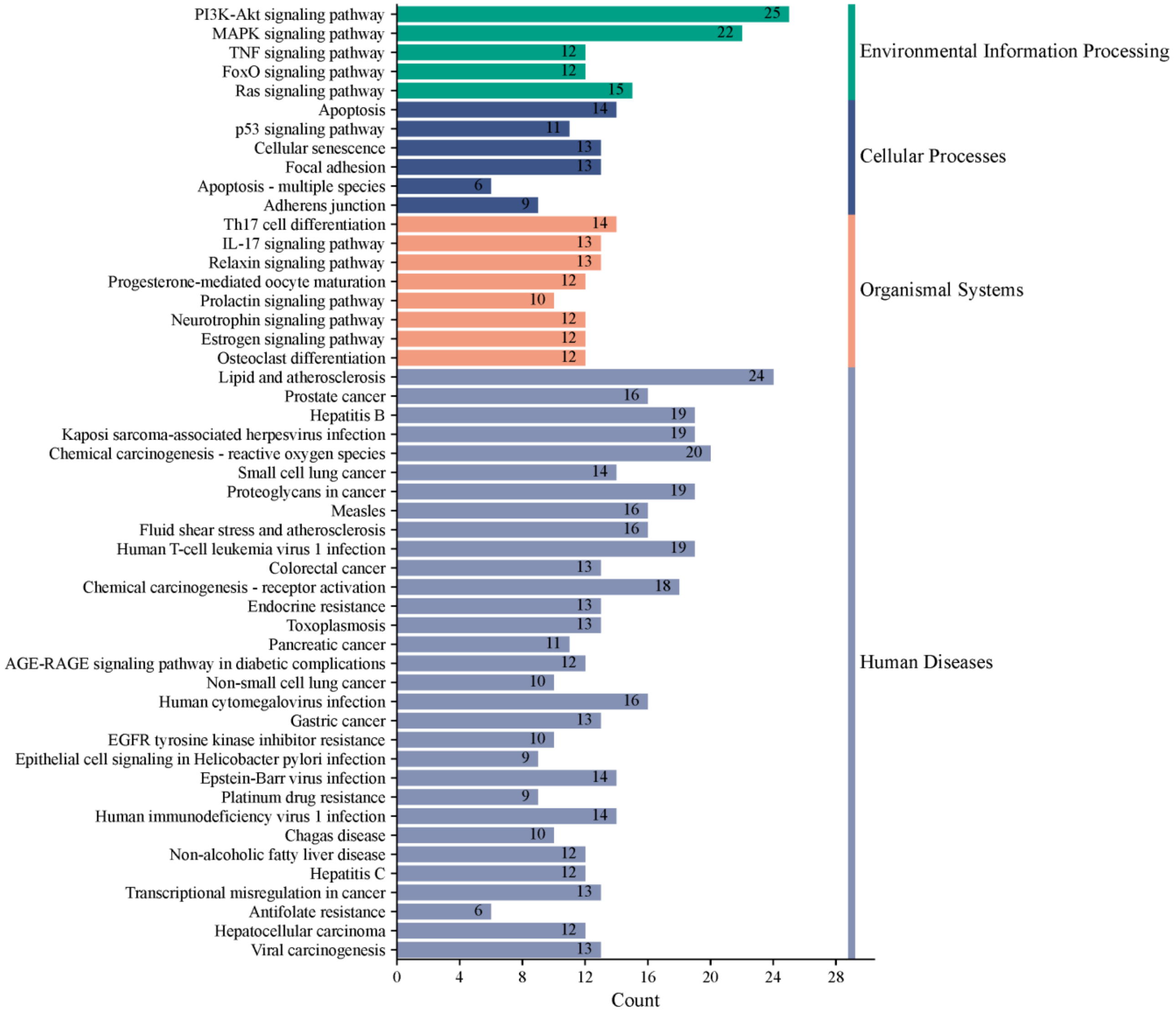

Supplementary Figure 2. Bar chart of taxonomic analysis of KEGG enrichment pathways.

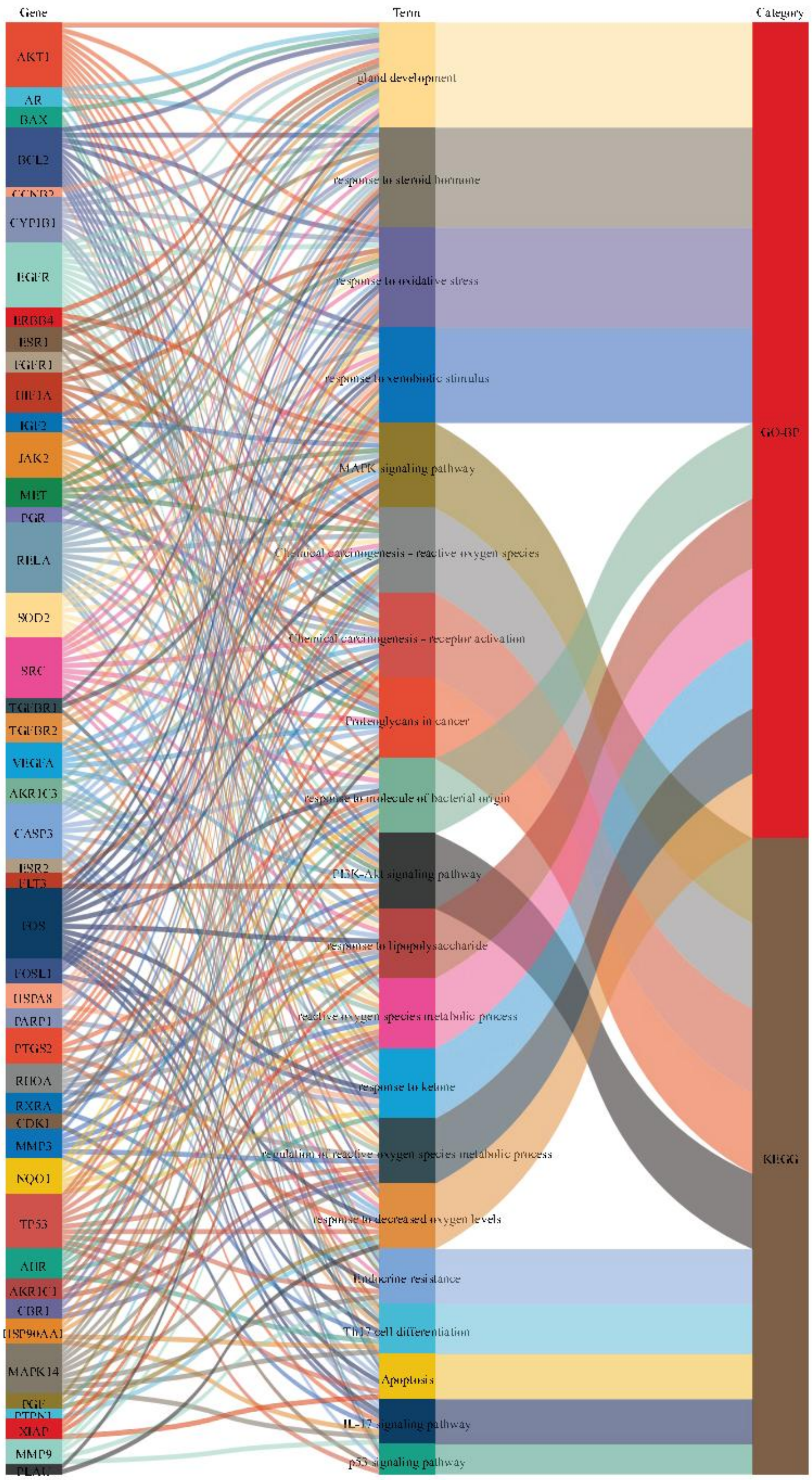
Gene
Term
Category
AKT1
AR
BAX
BCL2
CYP1B1
EGFR
ERBB4
ESR1
FGFR1
HIF1A
IGF2
JAK2
MET
PGR
RELA
SOD2
SRC
TGFBR1
TGFBR2
VEGFA
AKR1C3
CASP3
ESR2
FLT3
FOS
FOSL1
HSPA8
PARP1
PTGS2
RHOA
RXRA
CDK1
MMP3
NQO1
TP53
AHR
AKR1C1
CBR1
HSP90AA1
MAPK14
PGF
PTPN1
XIAP
MMP9
gland development
response to steroid hormone
response to oxidative stress
response to xenobiotic stimulus
MAPK signaling pathway
Chemical carcinogenesis - reactive oxygen species
Chemical carcinogenesis - receptor activation
Proteoglycans in cancer
response to molecule of bacterial origin
PI3K-Akt signaling pathway
response to lipopolysaccharide
reactive oxygen species metabolic process
response to ketone
regulation of reactive oxygen species metabolic process
response to decreased oxygen levels
Endocrine resistance
Th17 cell differentiation
Apoptosis
IL-17 signaling pathway
p53 signaling pathway
GO-BP
KEGG

Supplementary Figure 3. Sankey diagram. Sankey diagram of the distribution of the targets across key GO-BP and LUAD-related KEGG terms.

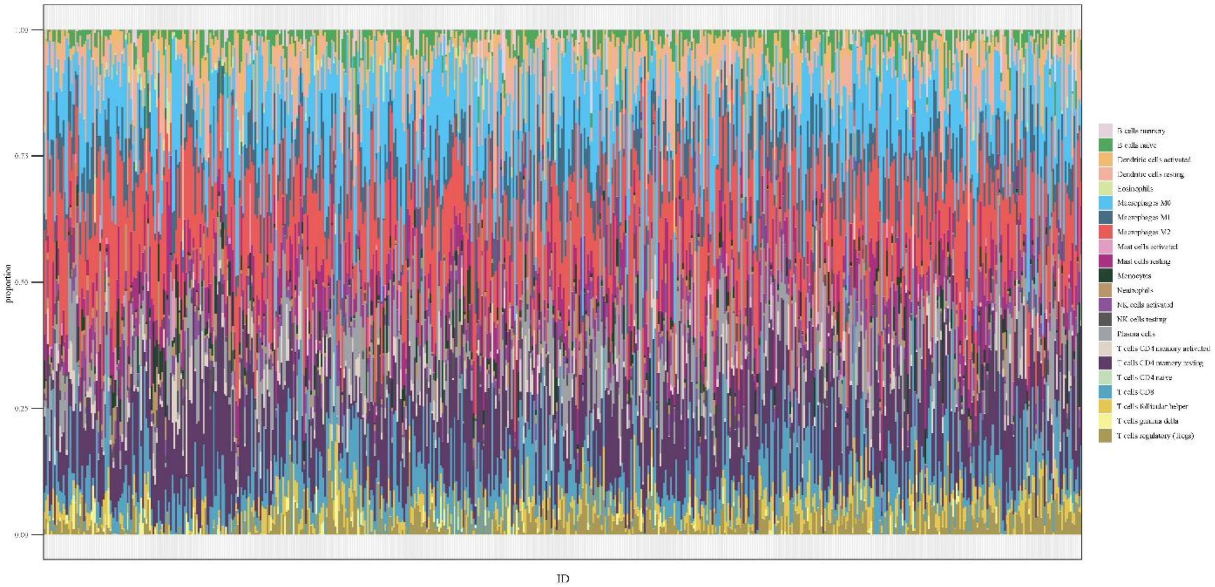

Supplementary Figure 4. Immune cell infiltration profiles estimated by CIBERSORT. Relative proportions of 22 immune cell types in each sample from the TCGA-LUAD RNA-seq dataset.

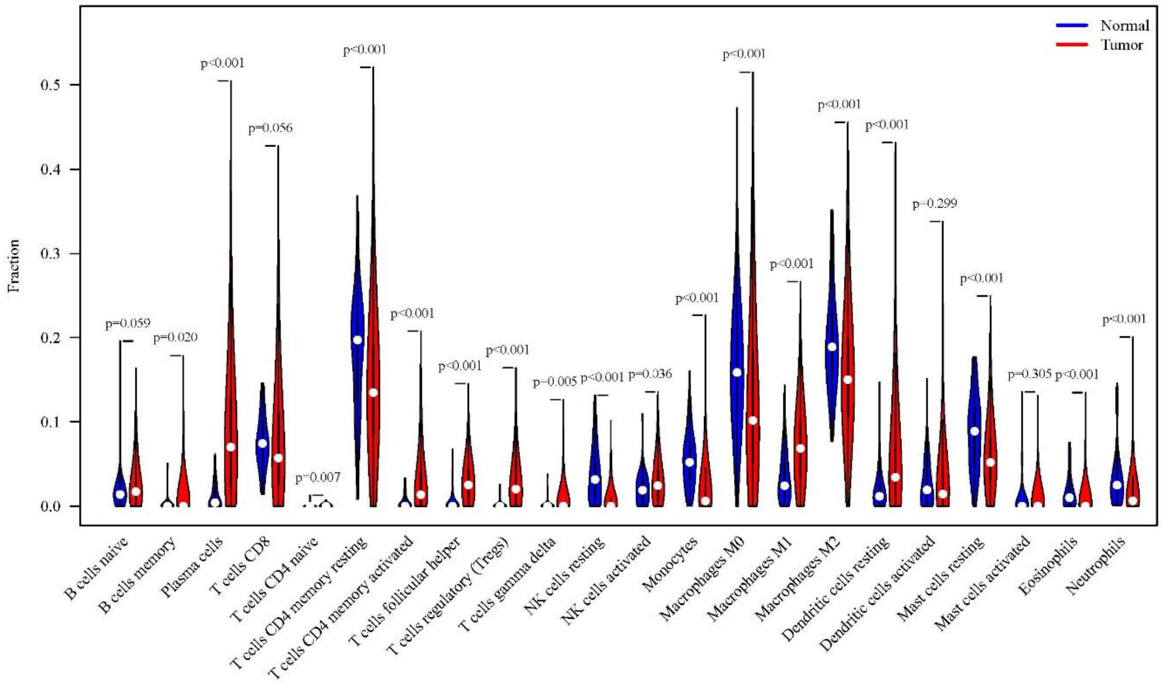

Supplementary Figure 5. Differences in immune cell infiltration between LUAD and normal lung tissues. Comparison of the infiltration levels of 22 immune cell types between LUAD tissues and normal controls based on CIBERSORT analysis.

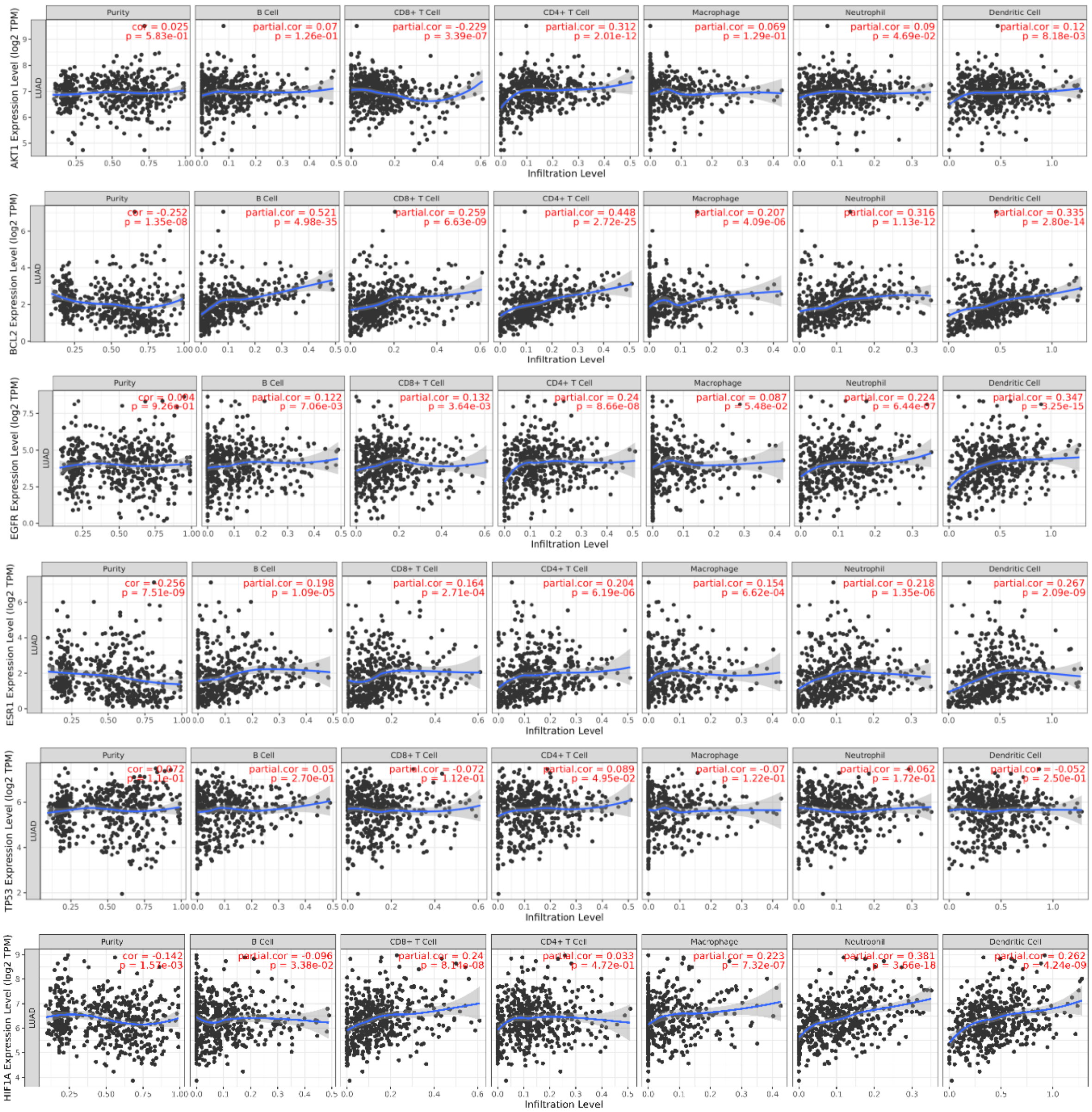

Supplementary Figure 6. Immune infiltration results based on TIMER. Immune infiltration levels of AKT1, BCL2, EGFR, ESR1, TP53 and HIF1A were analyzed using the TIMER algorithm.

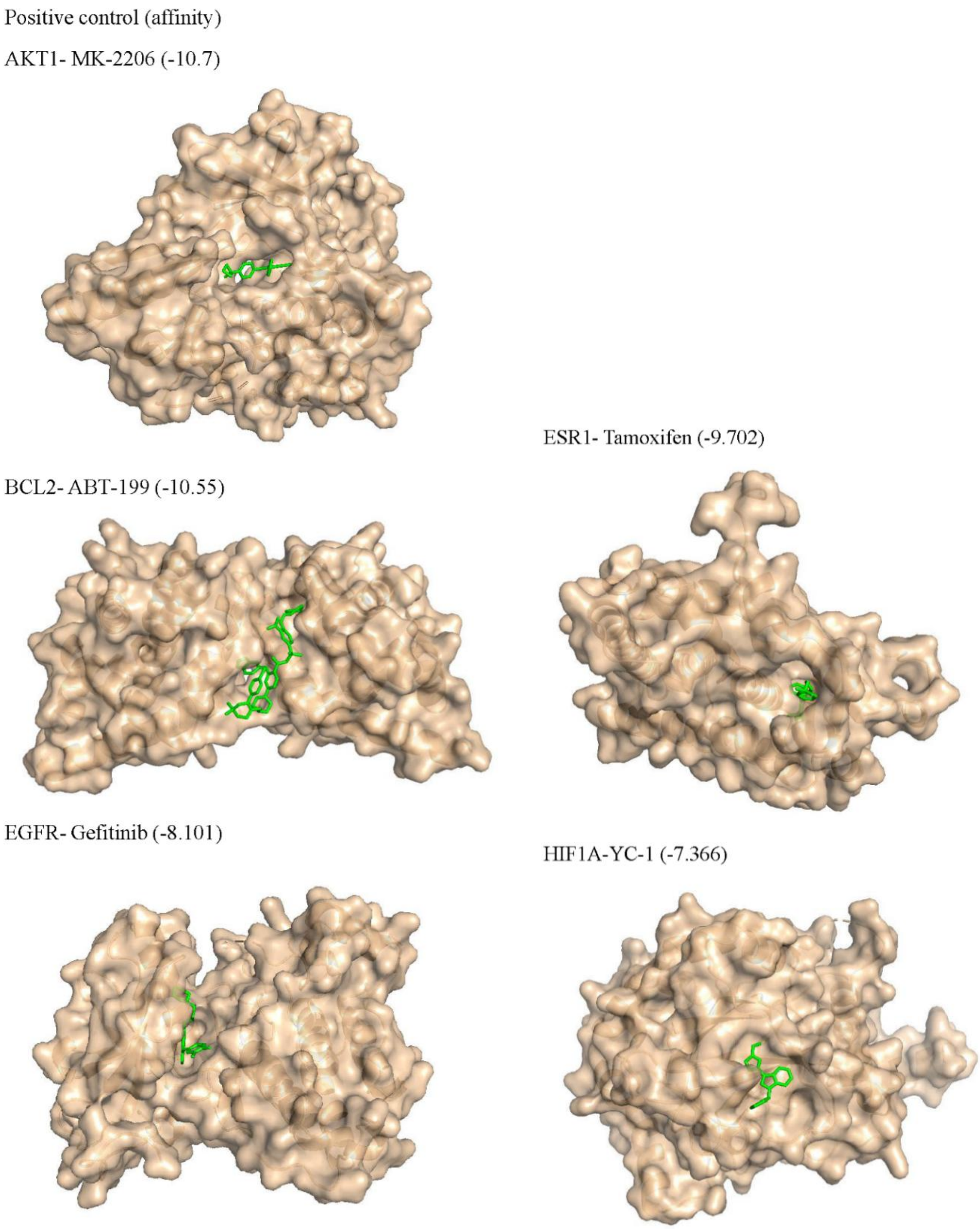

Supplementary Figure 7. Positive-control docking. Known inhibitors (MK-2206 for AKT1, ABT-199 for BCL2, gefitinib for EGFR, tamoxifen for ESR1, and YC-1 for HIF1A) were included as positive controls and docked using the same protocol. Binding energies(kcal/mol) are given in parentheses.

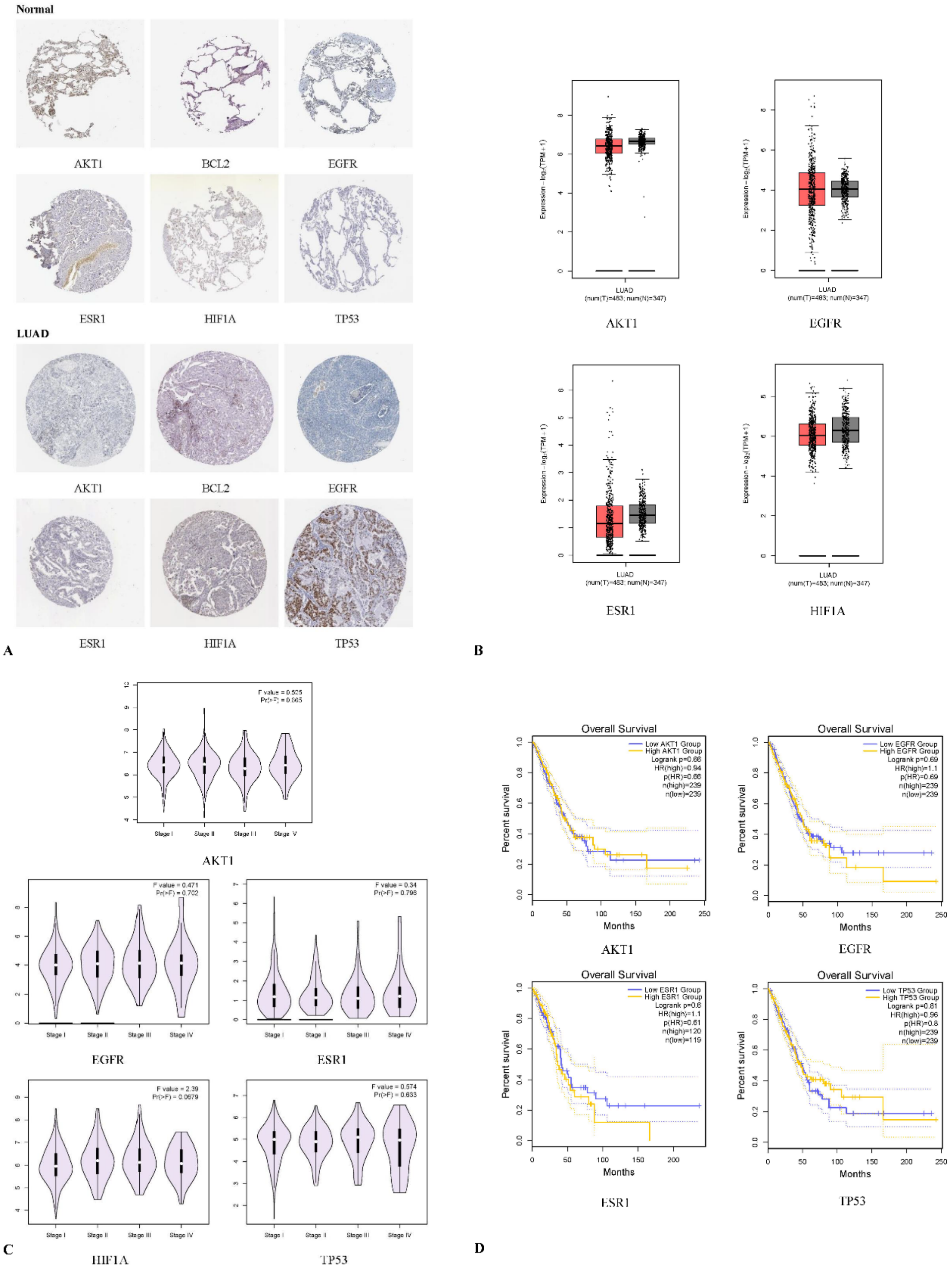

Supplementary Figure 8. Expression and prognostic analysis of hub genes. (A) The expression of hub targets at the protein levels in normal versus LUAD samples. (B) The expression of AKT1, EGFR, ESR1 and HIF1A at the transcriptome levels in normal versus LUAD samples. (C) The expression of AKT1, EGFR, ESR1, HIF1A and TP53 at different stages of LUAD. (D) Survival analysis of AKT1, EGFR, ESR1 and TP53 expression and LUAD prognosis.

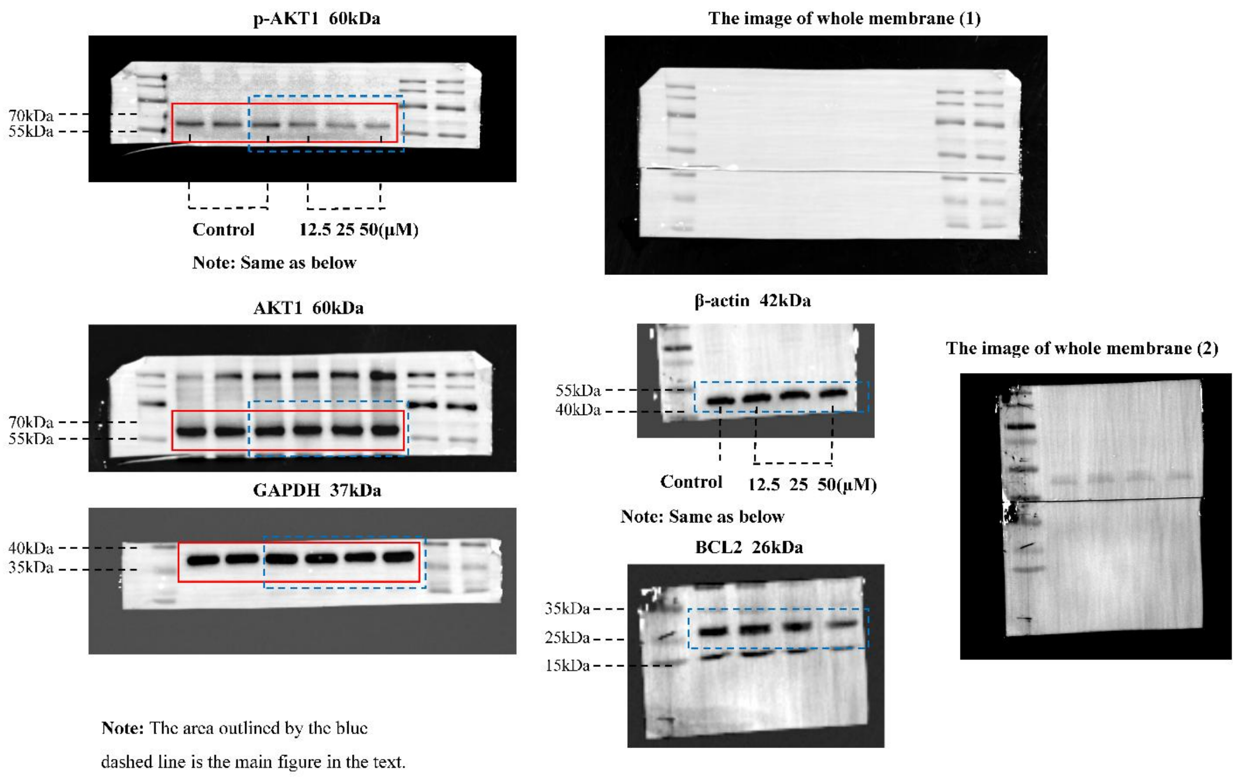

Supplementary Figure 9. Raw image. Raw images (western blot).
